# Supplementary material for: Lipopolysaccharide confinement in the bacterial outer membrane is governed by interactions within the conserved Lipid A anchor
Source: EMBO J. 2026 Feb 17;45(7):2338–69. doi: 10.1038/s44318-026-00711-5 (PMC13043748; doi:10.1038/s44318-026-00711-5)
Supplement: Supplementary file 1 — Table EV1 [file 44318_2026_711_MOESM1_ESM.docx]

**Table EV1:** Lateral diffusion of LPS and CirA receptor in the Gram-negative bacterial outer membrane characterized by SPT-TIRFM

| **Bacterial strain** | **Treatment** | **SPT probe** | **SPT imaging rate** (Hz) | **Number of tracks** | **D_2D_** ± **S.E.M.** (µm^2^/s) | **Confinement diameter** ± **S.E.M.** (µm) | **Type of lateral diffusion** |
| --- | --- | --- | --- | --- | --- | --- | --- |
| *E. coli* MG1655 | None | AF488-rough LPS | 30 | 156 | 0.0182 ± 0.000645 | 0.543 ± 0.00666 | confined |
| *E. coli* MG1655 | None | AF488-rough LPS | 67 | 85 | 0.0621 ± 0.00399 | 0.662 ± 0.0139 | confined |
| *E. coli* MG1655 | 100 mM EDTA | AF488-rough LPS | 30 | 118 | 0.0377 ± 0.00266 | 0.815 ± 0.0238 | confined |
|  |  |  | 30 | 31 | 0.153 ± 0.0216 | --- | free |
| *E. coli* MG1655 | 100 mM EGTA | AF488-rough LPS | 30 | 108 | 0.0373 ± 0.00297 | 0.748 ± 0.0200 | confined |
|  |  |  | 30 | 21 | 0.113 ± 0.0143 | --- | free |
| *E. coli* MG1655 | 300 mM urea | AF488-rough LPS | 30 | 154 | 0.0246 ± 0.00136 | 0.613 ± 0.0139 | confined |
| *E. coli* MG1655 | 300 mM urea | AF488-rough LPS | 67 | 107 | 0.101 ± 0.00884 | 0.880 ± 0.0389 | confined |
| *E. coli imp4213* | None | AF488-rough LPS | 30 | 104 | 0.0347 ± 0.00237 | 0.723 ± 0.0207 | confined |
|  |  |  | 30 | 49 | 0.0550 ± 0.00744 | --- | free |
| *E. coli imp4213* | None | AF488-rough LPS | 67 | 78 | 0.0865 ± 0.00672 | 0.774 ± 0.0272 | confined |
|  |  |  | 67 | 14 | 0.140 ± 0.0254 | --- | free |
| *E. coli* Δ*waaC* | None | AF488-deep rough LPS | 30 | 158 | 0.0182 ± 0.000722 | 0.542 ± 0.00617 | confined |
| *E. coli* Δ*waaC* | None | AF488-deep rough LPS | 67 | 144 | 0.0480 ± 0.00266 | 0.596 ± 0.00981 | confined |
| E. coli MG1655 | None | AF488-colicin Ia / CirA | 30 | 84 | 0.0178 ± 0.000623 | 0.540 ± 0.00665 | confined |
| *E. coli* MG1655 | None | AF488-colicin Ia / CirA | 67 | 105 | 0.0514 ± 0.00242 | 0.583 ± 0.00837 | confined |
| *E. coli* MG1655 | 100 mM EDTA | AF488-colicin Ia / CirA | 30 | 99 | 0.0454 ± 0.00340 | 0.846 ± 0.0229 | confined |
| *E. coli* MG1655 | 100 mM EDTA | AF488-colicin Ia / CirA | 67 | 40 | 0.140 ± 0.0155 | 1.10 ± 0.0624 | confined |
| *E. coli* MG1655 | 100 mM EGTA | AF488-colicin Ia / CirA | 30 | 74 | 0.0402 ± 0.00471 | 0.740 ± 0.0216 | confined |
| *E. coli* MG1655 | 100 mM EGTA | AF488-colicin Ia / CirA | 67 | 92 | 0.0620 ± 0.00406 | 0.704 ± 0.0238 | confined |
| *E. coli* MG1655 | 300 mM urea | AF488-colicin Ia / CirA | 30 | 107 | 0.0319 ± 0.00222 | 0.636 ± 0.0181 | confined |
| *E. coli* MG1655 | 300 mM urea | AF488-colicin Ia / CirA | 67 | 104 | 0.0672 ± 0.00515 | 0.667 ± 0.0201 | confined |
| *E. coli* ClearColi | Cultured with 4 mM Kdo sugar | AF488-colicin Ia / CirA | 30 | 78 | 0.0229 ± 0.00206 | 0.600 ± 0.0124 | confined |
| *E. coli* ClearColi | Cultured without Kdo sugar | AF488-colicin Ia / CirA | 30 | 90 | 0.0181 ± 0.000820 | 0.546 ± 0.00760 | confined |
