# Supplementary material for: Lipopolysaccharide confinement in the bacterial outer membrane is governed by interactions within the conserved Lipid A anchor
Source: EMBO J. 2026 Feb 17;45(7):2338–69. doi: 10.1038/s44318-026-00711-5 (PMC13043748; doi:10.1038/s44318-026-00711-5)
Supplement: Supplementary file 2 — Appendix [file 44318_2026_711_MOESM2_ESM.pdf]

APPENDIX

Lipopolysaccharide confinement in the bacterial outer membrane is governed by interactions within the conserved Lipid A anchor

Joe Nabarro[1,2,5], Rosalyn M. Leaman[1,5], Samuel Lenton[1], Leonore Mantion[1], Richard J. Spears[2], Mark C. Coles[3], Dmitri O. Pushkin[4], Martin A. Fascione[2,6] and Christoph G. Baumann[1,6]

Affiliations

[1] Department of Biology, University of York, York YO10 5DD, United Kingdom

[2] Department of Chemistry, University of York, York YO10 5DD, United Kingdom

[3] Kennedy Institute of Rheumatology, Nuffield Department of Orthopaedics, Rheumatology and Musculoskeletal Science, University of Oxford, Oxford OX3 7FY, United Kingdom

[4] Department of Mathematics, University of York, York YO10 5DD, United Kingdom

[5] These authors contributed equally

[6] Co-corresponding authors

Table of Contents

	Page		Page
Appendix Figure S1	2	Appendix Table S1	12
Appendix Figure S2	3	Appendix Table S2	13
Appendix Figure S3	4	Appendix Table S3	13
Appendix Figure S4	5	Appendix Table S4	14
Appendix Figure S5	6	Appendix Table S5	15
Appendix Figure S6	7	Appendix Table S6	15
Appendix Figure S7	8	Appendix Table S7	15
Appendix Figure S8	9	Appendix Table S8	15
Appendix Figure S9	10	Appendix Table S9	16
Appendix Figure S10	11	Appendix Table S10	16
Appendix Figure S11	11	Appendix Table S11	16
		Appendix Table S12	16
		Appendix Table S13	17
		Appendix Table S14	17
		Appendix Table S15	17
		Appendix Table S16	18
		Appendix Table S17	18

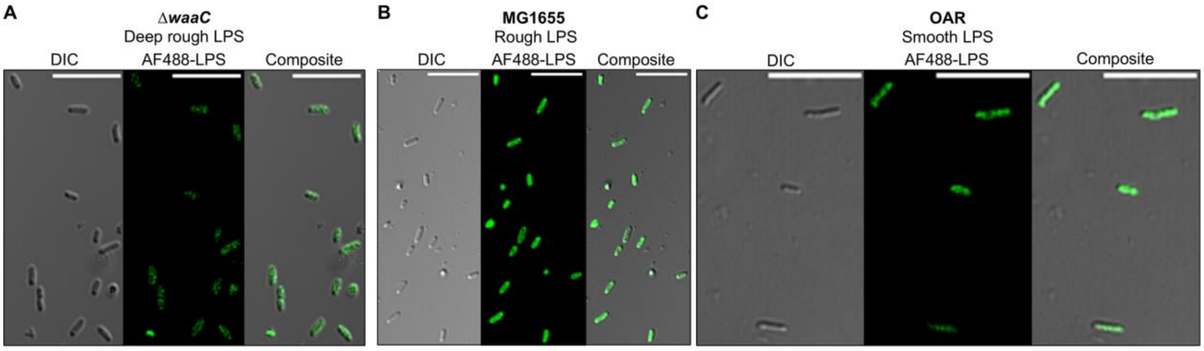

Appendix Figure S1: Wide-field confocal fluorescence microscopy images showing efficient *in vivo* fluorescent labeling of bacterial cells with different LPS glycoforms. (**A**) *E. coli* Δ*waaC* cells producing deep rough LPS, (**B**) *E. coli* MG1655 cells producing rough LPS and (**C**) O-antigen restored (OAR) *E. coli* DFB1655 cells producing smooth LPS. The different LPS glycoforms were labeled using a two-step metabolic / bio-orthogonal labeling approach involving i) metabolic labeling of LPS using an azide functionalized Kdo-analogue followed by ii) *in situ* AF488-alkyne conjugation via Cu(I)-catalyzed azide-alkyne cycloaddition. Scale bars: 10 µm.

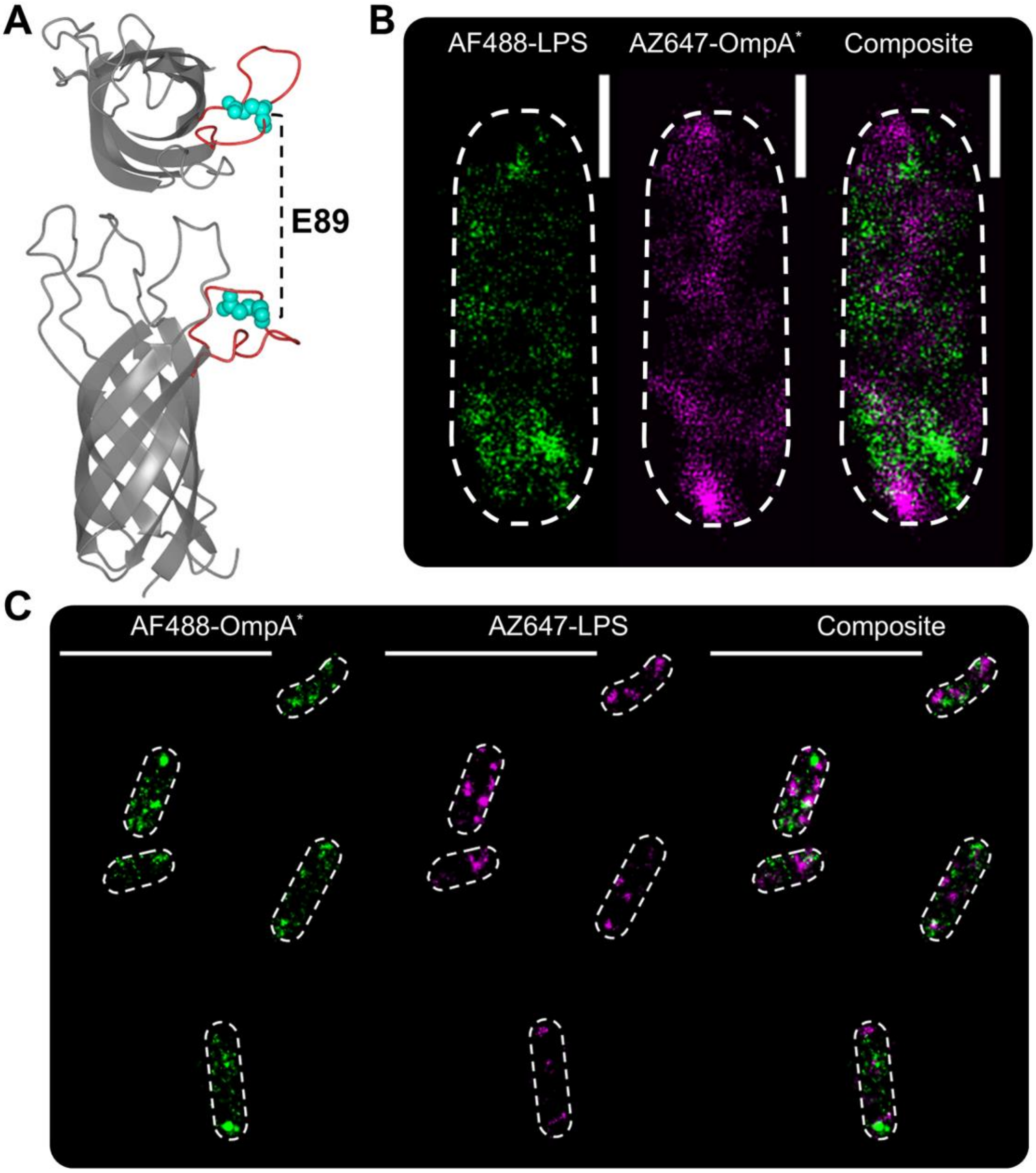

Appendix Figure S2: *In situ* fluorescent labeling of OmpA using amber stop codon suppression and genetic code expansion followed by fluorophore conjugation via CuAAC enabled simultaneous visualization of discrete OMP and LPS distributions in the OM of single cells by two-color dSTORM. (**A**) AlphaFold2 generated 3D structure of *E. coli* OmpA highlighting the site of *N*-propargyl-L-lysine incorporation which replaces a glutamic acid residue (E89) in extracellular loop 2. (**B**) Two-color single-cell dSTORM images of *E. coli* Δ*ompA* cells producing recombinant OmpA* and incorporating Kdo-azide modified LPS. In the single-cell view, the separate images show the discrete positions of AF488-labeled LPS molecules and AZ647-labeled OmpA* proteins in the OM, and the composite image shows that the spatial distribution of these two OM components is heterogeneous. (**C**) Two-color wide-field dSTORM images of multiple *E. coli* Δ*ompA* cells producing recombinant OmpA* and incorporating Kdo-azide modified LPS after dual fluorescent labeling. The images of AF488-OmpA* and AZ647-LPS in the OM show that the spatial distribution of the LPS- and OMP-rich regions varies between cells, and does not depend on the fluorophore used to localize LPS and OmpA*. Scale bars in wide-field view = 5.0 μm. Scale bars in single-cell view = 0.5 μm.

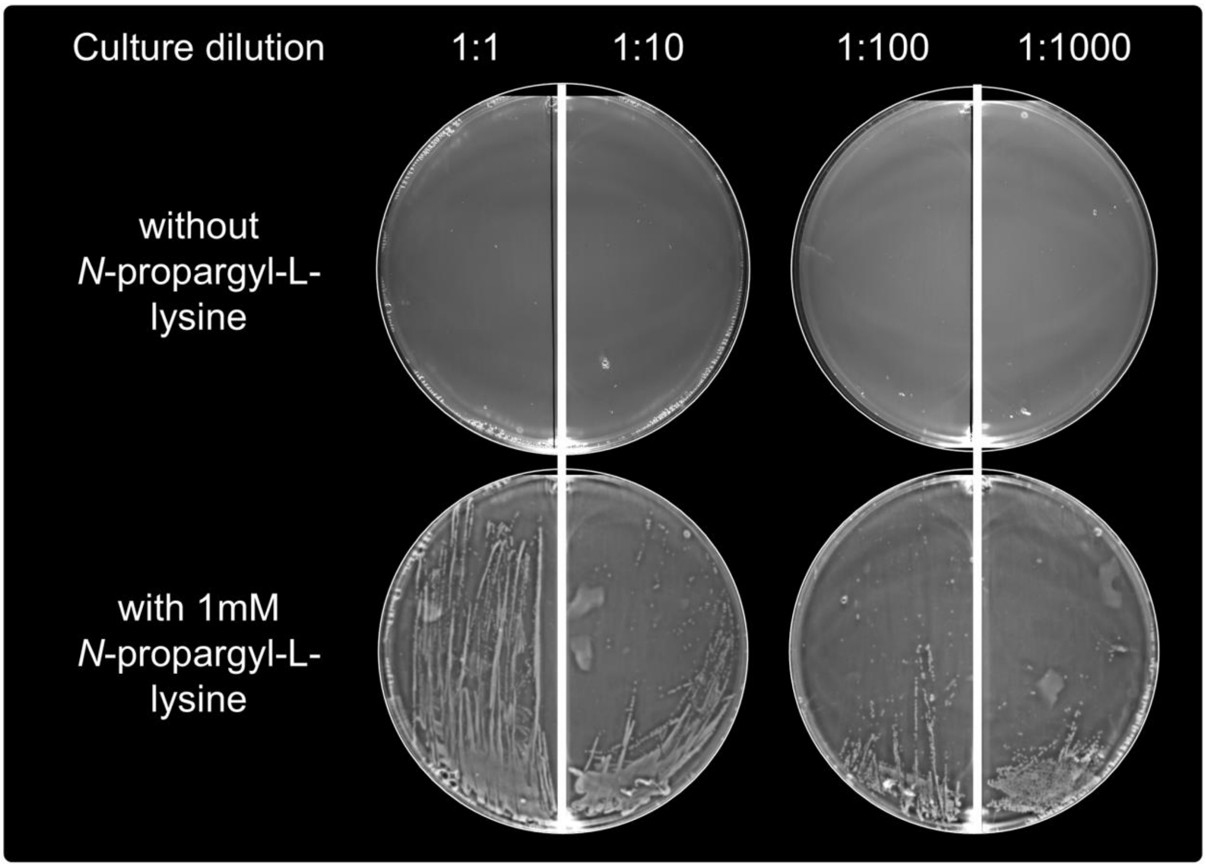

Appendix Figure S3: Incorporation of *N*-propargyl-L-lysine in extracellular loop 2 of *E. coli* OmpA to replace a glutamic acid residue (E89) via amber stop codon suppression and genetic code expansion produces functional recombinant protein. Recombinant OmpA* production was done in the *E. coli* BW25113 Δ*ompA* strain for which growth was inhibited on LB-Miller agar plates containing 0.5% (w/v) SDS and 1 mM EDTA. Cultures of the *E. coli* BW25113 Δ*ompA* cells co-transformed with the pBAD-ompA* and pEVOL-pylRS plasmids were spread on the selective plates at four different dilutions (1:1, 1:10, 1:100 and 1:1000), and the plates were incubated overnight at 37°C. The transformed strain did not grow on LB-Miller / 0.5% (w/v) SDS / 1 mM EDTA agar plates without *N*-propargyl-L-lysine added (top row) but did grow on the same plates supplemented with 1 mM *N*-propargyl-L-lysine (bottom row). The SDS and EDTA induced growth inhibition phenotype was not observed in the *ompA** expressing *E. coli* Δ*ompA* strain grown with 1 mM *N*-propargyl-L-lysine, therefore the recombinant OmpA* protein is able to substitute for wild-type OmpA.

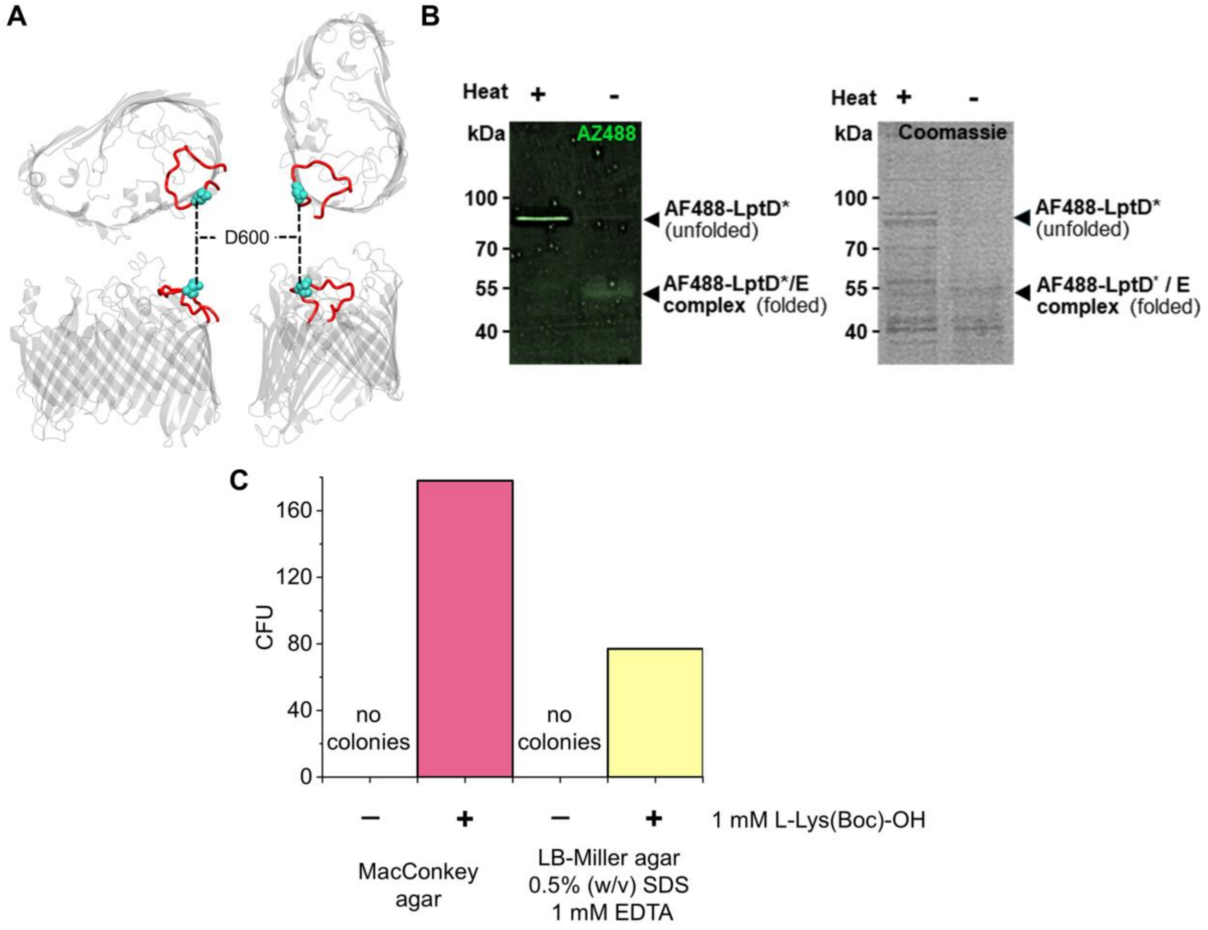

Appendix Figure S4: Incorporation of ε-(tert-Butoxycarbonyl)-L-lysine or *N*-propargyl-L-lysine into extracellular loop 9 of *E. coli* LptD via amber stop codon suppression and genetic code expansion. (**A**) LptD crystal structure [PDB: 4HRB] highlighting loop 9 (red) and position of D600 where the site of amber stop codon mutation was introduced and the ncAA was incorporated. (**B**) Production of functional LptD was regulated by addition of the following inert ncAAs: ε-(tert-Butoxycarbonyl)-L-lysine (L-Lys(Boc)-OH) allowed biochemical control over full-length LptD translation, and clickable L-Lys(Propargyl)-OH enabled subsequent fluorescent visualisation of protein bands for LptD* and the native LptD*/LptE complex by SDS-PAGE. SDS-PAGE was used to characterize cell lysates containing folded (unheated, lanes with 'minus' sign) or denatured (heat treated, lanes with 'plus' sign) fluorescently labeled recombinant LptD*. *N*-propargyl-L-lysine incorporation into LptD* followed by *in situ* fluorescent labeling with AF488-alkyne via CuAAC does not disrupt native complex formation with LptE demonstrating ncAA-LptD* is functional. Heat denaturation of the cell lysate disrupted the LptD* / LptE complex and resulted in the reduced mobility of denatured LptD* during SDS-PAGE. The SDS-PAGE gel was imaged with 488 nm laser excitation to view fluorescently labeled LptD* band (left), and with visible light after Coomassie brilliant blue staining to view all protein bands present (right). (**C**) Production of L-Lys(Boc)-OH containing LptD* in *imp4213* cells co-transformed with pBAD-lptD* and pEVOL-pylRS plasmids restored wild-type OM barrier function. This made *imp4213* cells co-transformed with pBAD-lptD* and pEVOL-pylRS plasmids tolerant to normally lethal concentrations of bile salts (MacConkey agar) and detergent / chelator (0.5% (w/v) SDS, 1 mM EDTA) when grown with 1 mM L-Lys(Boc)-OH. Colony forming units (CFU) for a representative growth screening experiment are provided.

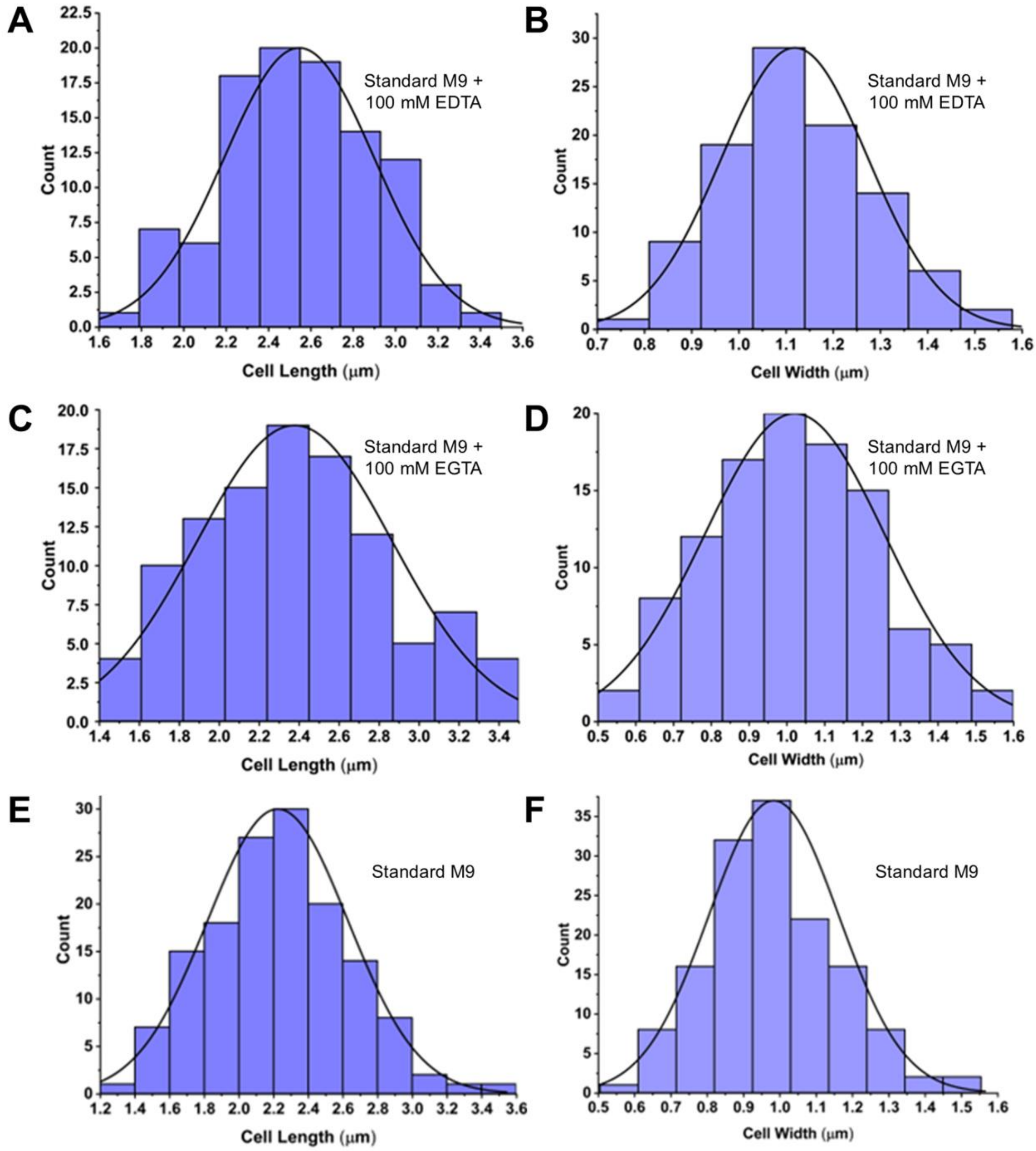

Appendix Figure S5: Morphology of *E. coli* BW25113 cells was not altered by EDTA and EGTA treatments. (**A**) Distributions of cell length (mean ± S.D. = 2.55 ± 0.354 µm) and (**B**) width (mean ± S.D. = 1.12 ± 0.156 µm) obtained for *E. coli* BW25113 cells (n = 101) after 30 min treatment with 100 mM EDTA in standard M9 CDM at 20°C. (**C**) Distributions of cell length (mean ± S.D. = 2.38 ± 0.490 µm) and (**D**) width (mean ± S.D. = 1.02 ± 0.239 µm) obtained for *E. coli* BW25113 cells (n = 108) after 30 min treatment with 100 mM EGTA in standard M9 CDM at 20°C. (**E**) Distributions of cell length (mean ± S.D. = 2.23 ± 0.406 µm) and (**F**) width (mean ± S.D. = 0.984 ± 0.179 µm) obtained for untreated *E. coli* BW25113 cells (n = 144) in standard M9 CDM are provided for comparison. The cell dimensions were measured directly from pre- and post-FRAP wide-field DIC microscopy images acquired at 20°C across three independent experiments. Non-linear regression of each histogram was done using a standard Gaussian function (solid lines) in OriginPro.

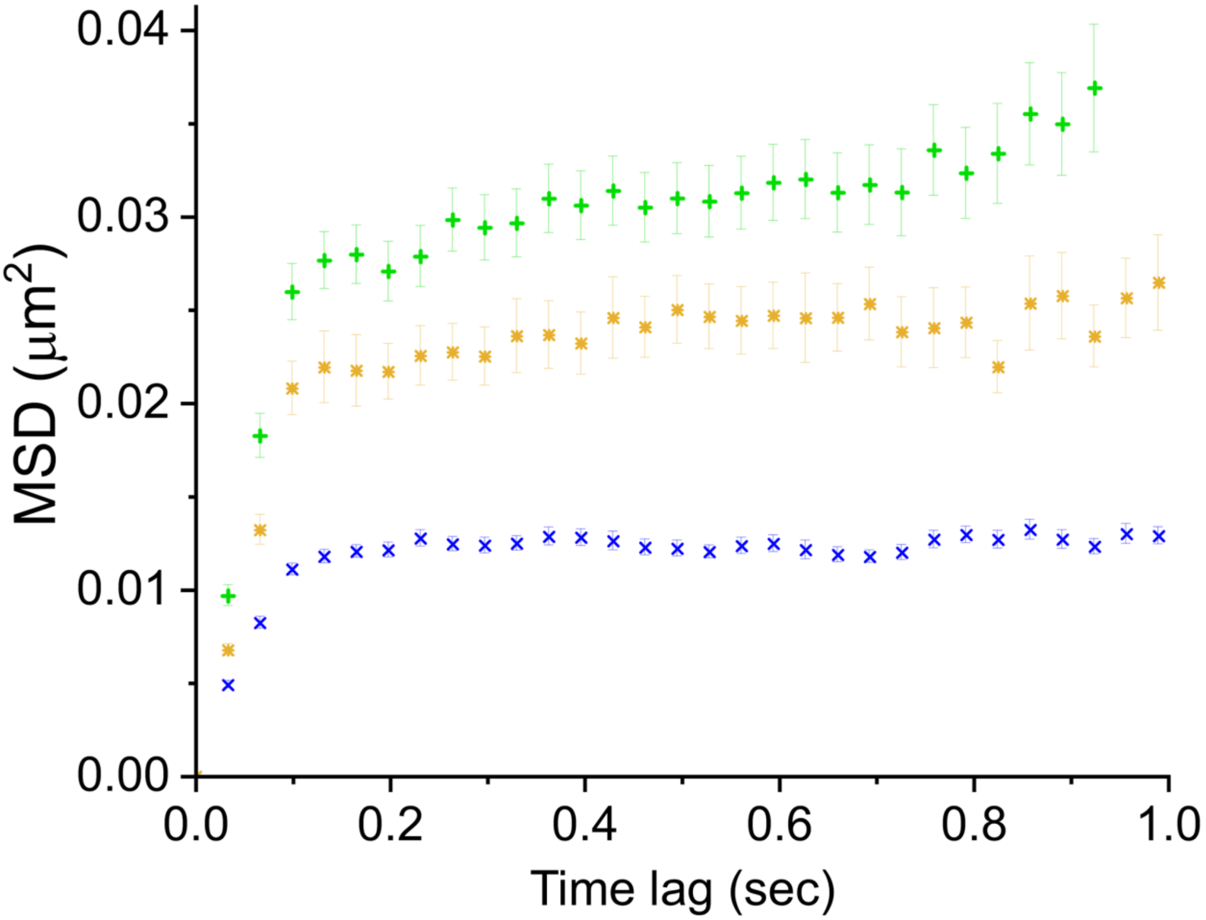

Appendix Figure S6: **Effect of chelator treatments on the lateral diffusion of OMPs (CirA receptor) as measured on *E. coli* MG1655 cells by SPT-TIRFM.** MSD was calculated for single AF488-labeled colicin Ia / CirA receptor complexes that could be tracked for at least 0.9 s before photobleaching (error is reported as S.E.M.). All total internal reflection fluorescence microscopy video data was collected at 30 Hz from a minimum of three experimental replicates using 488 nm laser illumination. The MSD value for all OMP complexes on untreated *E. coli* MG1655 cells (blue **×**, n = 84 tracks) and on cells treated with 100 mM EDTA (green **+**, n = 99 tracks) or 100 mM EGTA (orange ■, n = 74 tracks) approached an asymptotic value which was consistent with confined lateral diffusion. However, the level of confinement for OMP complexes was reduced in the chelator treated cells relative to the untreated cells. Linear regression of the MSD for the first 4 time delays yielded D_{2D} ≈ 0.0454 $\mu m^2/s$ and D_{2D} ≈ 0.0402 $\mu m^2/s$, respectively, for the OMP complexes in EDTA- and EGTA-treated cells. These D_{2D} values were increased relative to the value for untreated cells (~0.0178 $\mu m^2/s$). The reduction in membrane confinement induced by chelator treatments was accompanied by a 2- to 3-fold increase in the apparent rate of lateral diffusion.

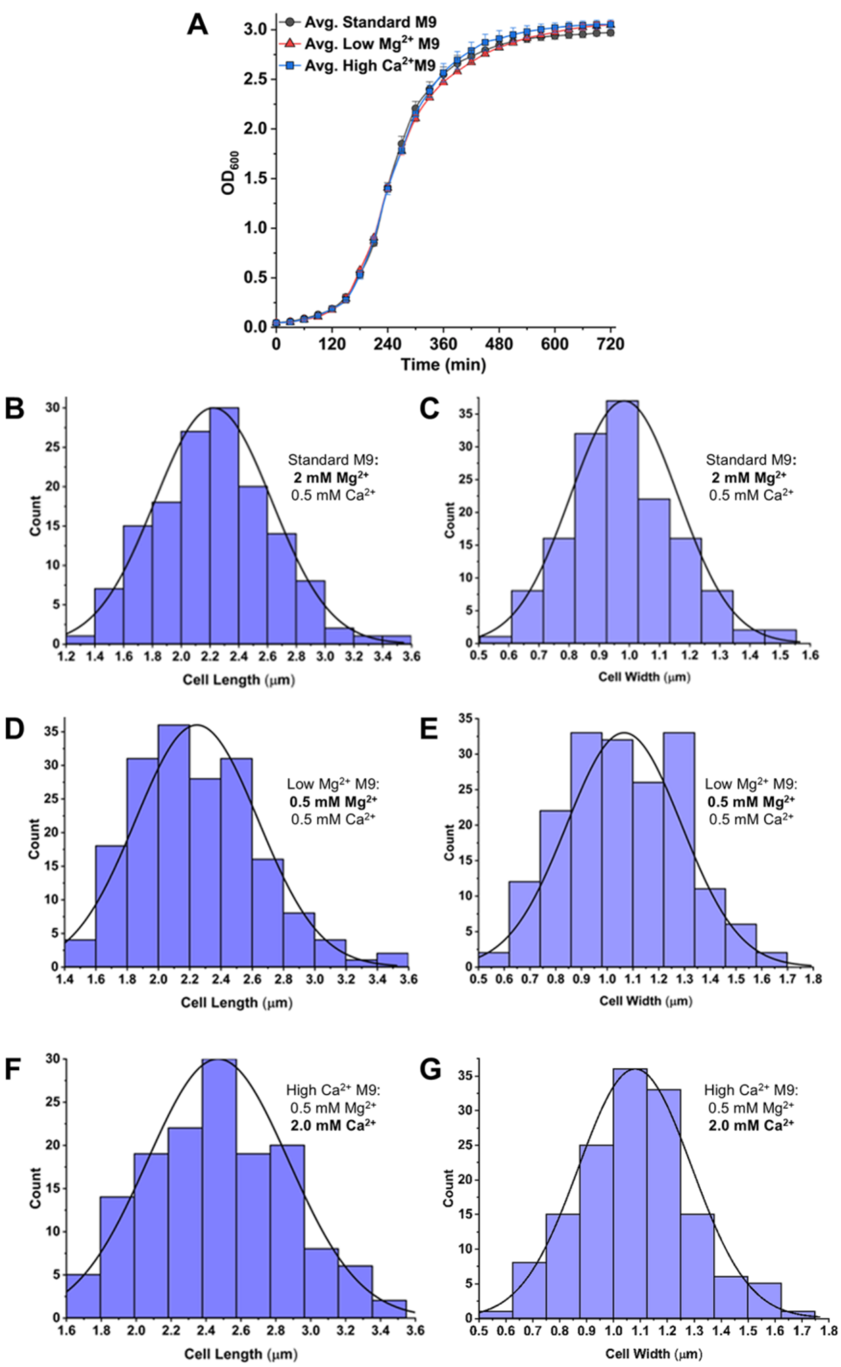

Appendix Figure S7: Growth and morphology of *E. coli* BW25113 cells were not affected by varying Mg^{2+} and Ca^{2+} ion concentrations in the M9 CDM. (**A**) Average growth curves (n = 3) for *E. coli* BW25113 strain cultured at 37°C in standard M9 CDM (2 mM Mg^{2+}, 0.5 mM Ca^{2+}), low Mg^{2+} M9 CDM (0.5 mM Mg^{2+}, 0.5 mM Ca^{2+}), and high Ca^{2+} M9 CDM (0.5 mM Mg^{2+}, 2.0 mM Ca^{2+}). Variations in divalent cation concentrations did not affect bacterial growth rates. (**B**) Distributions of cell length (mean ± S.D. = 2.23 ± 0.406 μm) and (**C**) width (mean ± S.D. = 0.984 ± 0.179 μm) obtained for *E. coli* BW25113 cells (n = 144) in standard M9 CDM. (**D**) Distributions of cell length (mean ± S.D. = 2.25 ± 0.393 μm) and (**E**) width (mean ± S.D. = 1.07 ± 0.223 μm) obtained for *E. coli* BW25113 cells (n = 179) in low Mg^{2+} M9 CDM. (**F**) Distributions of cell length (mean ± S.D. = 2.47 ± 0.405 μm) and (**G**) width (mean ± S.D. = 1.08 ± 0.210 μm) obtained for *E. coli* BW25113 cells (n = 145) in high Ca^{2+} M9 CDM. The cell dimensions were measured directly from pre- and post-FRAP wide-field DIC microscopy images acquired at 20°C across three independent experiments. Non-linear regression of each histogram was done using a standard Gaussian function (solid lines) in OriginPro.

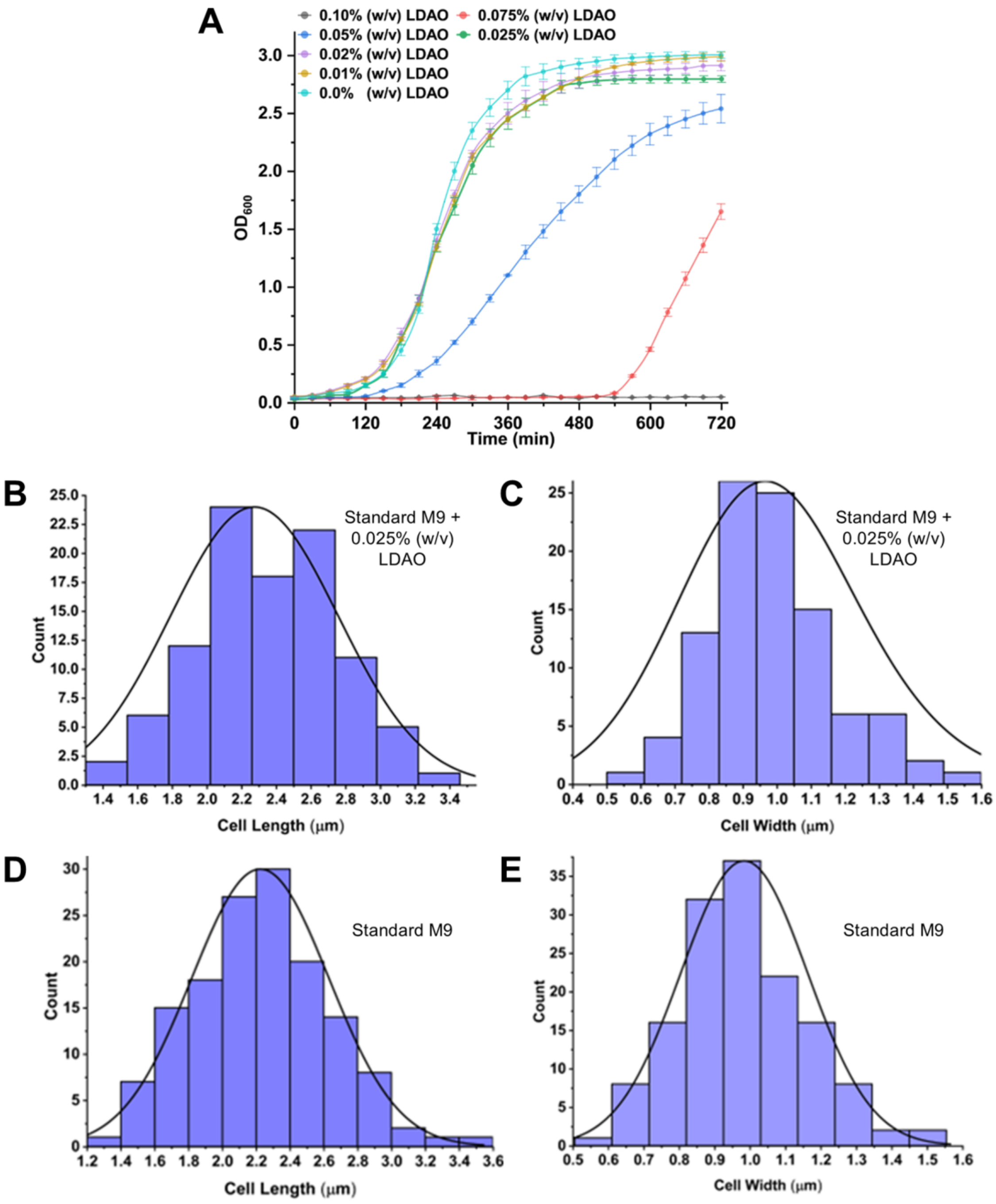

Appendix Figure S8: Growth and morphology of *E. coli* BW25113 cells were not altered by adding 0.025% (w/v) LDAO to the standard M9 CDM. (**A**) Average growth curves (n = 3) for *E. coli* BW25113 strain cultured at 37°C in standard M9 CDM with increasing concentrations of LDAO detergent. 0.025% (w/v) LDAO was the highest detergent concentration that maintained growth rates equivalent to cultures without detergent added. (**B**) Distributions of cell length (mean ± S.D. = 2.27 ± 0.485 μm) and (**C**) width (mean ± S.D. = 1.11 ± 0.496 μm) obtained for *E. coli* BW25113 cells (n = 107) after 30 min treatment with 0.025% (w/v) LDAO in standard M9 CDM at 20°C. (**D**) Distributions of cell length (mean ± S.D. = 2.23 ± 0.406 μm) and (**E**) width (mean ± S.D. = 0.984 ± 0.179 μm) obtained for *E. coli* BW25113 cells (n = 144) in standard M9 CDM. The cell dimensions were measured directly from pre- and post-FRAP wide-field DIC microscopy images acquired at 20°C across three independent experiments. Non-linear regression of each histogram was done using a standard Gaussian function (solid lines) in OriginPro.

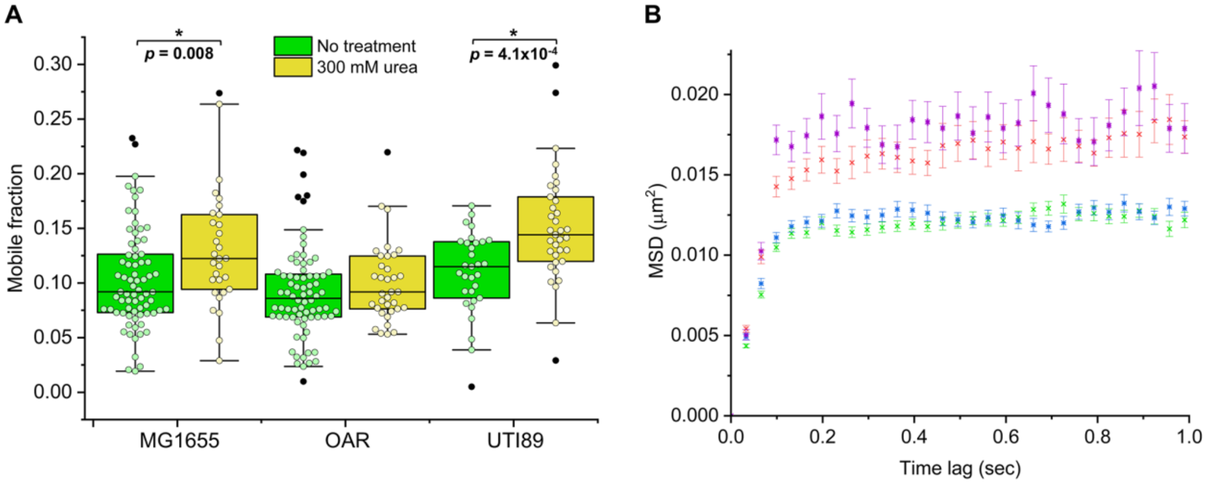

Appendix Figure S9: Treatment of bacterial cells with a non-lethal concentration of chaotropic agent (300 mM urea) induced a minor increase in lateral mobility of LPS and OMPs. (**A**) Effect of 300 mM urea (a concentration comparable to that found in human urine) on LPS mobile fractions in the OM of *E. coli* MG1655, *E. coli* DFB1655 O-antigen restored (OAR) and uropathogenic *E. coli* UTI89 cells measured via FRAP. MG1655: no treatment (green box), median = 0.092 (n = 74); 300 mM urea treatment (yellow box), median = 0.122 (n = 30). OAR: no treatment (green box), median = 0.088 (n = 75); 300 mM urea treatment (yellow box), median = 0.092 (n = 35). UTI89: no treatment (green box), median = 0.115 (n = 31); 300 mM urea treatment (yellow box), median = 0.141 (n = 35). Significant increases in the LPS mobile fraction distribution were observed for the urea treatment of MG1655 cells (by Mann-Whitney test, $p = 0.0082$) and UTI89 cells (by Mann-Whitney test, $p = 4.105 \times 10^{-4}$). Whiskers represent maximum and minimum values, and each colored dot represents an individual measurement collected over at least three experimental replicates. Dark grey dots outside whisker bounds are classified as outliers. (**B**) Mean-squared displacement (MSD) was calculated for single AF488-labeled LPS molecules and single AF488-labeled colicin Ia / CirA receptor complexes that could be tracked for at least 1 s before photobleaching (error is reported as S.E.M.). All total internal reflection fluorescence microscopy video data was collected at 30 Hz from a minimum of three experimental replicates using 488 nm laser illumination. The MSD for LPS molecules and the CirA receptor on untreated *E. coli* MG1655 cells and 300 mM urea treated cells approached an asymptotic value which was consistent with confined lateral diffusion. Treatment of cells with this chaotropic agent produced no measurable difference in the asymptotic MSD value relative to untreated cells for LPS and the CirA receptor. Linear regression of the MSD for the first 4 time delays yielded $D_{2D} \approx 0.0182\ \mu m^2/s$ (green **×**, n = 156 tracks) and $D_{2D} \approx 0.0246\ \mu m^2/s$ (red **×**, n = 154 tracks) for LPS on untreated and treated cells, respectively. Linear regression of the MSD for the same time delays yielded $D_{2D} \approx 0.0178\ \mu m^2/s$ (blue **✳**, n = 84 tracks) and $D_{2D} \approx 0.0319\ \mu m^2/s$ (purple **✳**, n = 107 tracks) for the CirA receptor on untreated and treated cells, respectively. The SPT data demonstrate that disruptions to the structure of the oligosaccharide domain induced by 300 mM urea cause a small reduction in the overall lateral confinement of LPS and CirA receptor, which is accompanied by a < 2-fold increase in the apparent rate of lateral diffusion for both LPS and the CirA receptor.

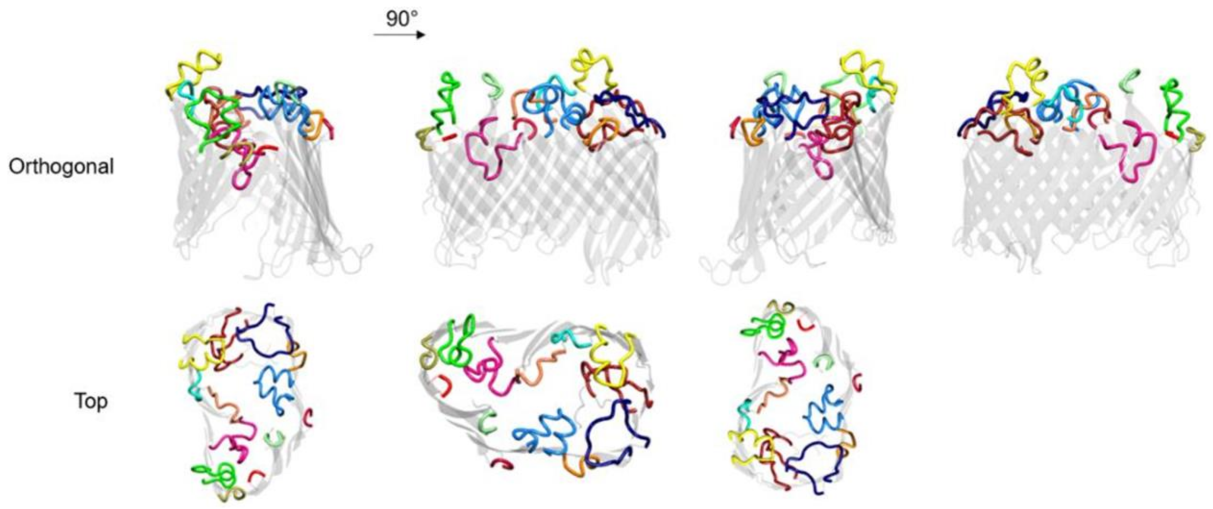

Appendix Figure S10. *Escherichia coli* K-12 LptD crystal structure [PDB: 4RHB] highlighting extracellular loops investigated for possible ncAA incorporation via genetic code expansion (GCE).

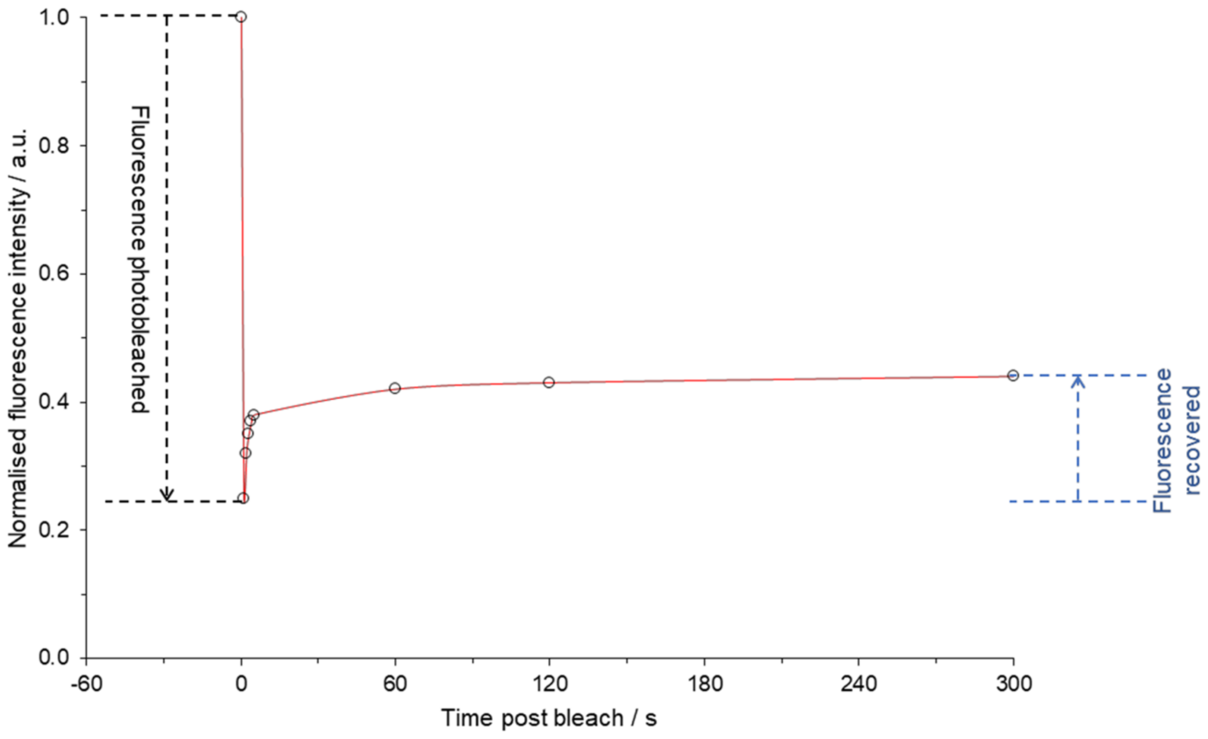

Appendix Figure S11. Example double-normalized FRAP recovery curve.

Appendix Table S1: Two-sided Mann-Whitney U-test statistics from comparison of FRAP-derived mobile fraction distributions

Population #1	Population #2	$n_{[\#1]}$	$n_{[\#2]}$	*p*	U	Z	Fig. ref.
AF594-PG	AF488-LPS	30	69	0.60	1177	0.5	1C
AF488-LPS	GFP-TolA	74	36	4.4 x 10^{-17}	2590	8.4	1C
AF594-PG	GFP-TolA	30	36	5.1 x 10^{-12}	1050	6.9	1C
MG1655	Δ*waaC*	69	49	0.2	1926	1.3	1F
MG1655	OAR	69	74	0.1	2973	1.5	1F
Δ*waaC*	OAR	49	74	0.7	1772	-0.3	1F
MG1655	Δ*lpp*	74	47	3.4 x 10^{-7}	1330	-5.1	3D
MG1655	*imp4213*	74	49	1.5 x 10^{-15}	283	-7.8	3D
MG1655	*imp4213* pBAD-lptD*	74	30	0.080	1301	1.8	3D
imp4213	*imp4213* pBAD-lptD*	49	30	5.9 x 10^{-12}	1354	6.8	3D
Δ*waaC* No treatment	Δ*waaC* 10 mM EGTA	49	39	8.8 x 10^{-3}	662	-2.9	4B
Δ*waaC* No treatment	Δ*waaC*<br>10 mM EDTA	49	48	2.1 x 10^{-19}	35	-9.0	4B
Δ*waaC*<br>No treatment	Δ*waaC*<br>0.025% (w/v) LDAO	49	30	1.2 x 10^{-13}	0	-7.42	4B
MG1655<br>No treatment	MG1655<br>100 mM EGTA	74	35	2.1 x 10^{-9}	372	-6.0	4B
MG1655<br>No treatment	MG1655<br>100 mM EDTA	74	31	3.3 x 10^{-13}	110	-7.3	4B
MG1655<br>No treatment	MG1655<br>0.025% (w/v) LDAO	74	37	1.2 x 10^{-15}	89	-8.0	4B
OAR<br>No treatment	OAR<br>100 mM EGTA	74	32	9.0 x 10^{-6}	547	-4.4	4B
OAR<br>No treatment	OAR<br>100 mM EDTA	74	37	1.6 x 10^{-7}	540	-5.2	4B
OAR<br>No treatment	OAR<br>0.025% (w/v) LDAO	74	35	2.6 x 10^{-5}	657	-4.2	4B
Δ*waaC*<br>10 mM EGTA	Δ*waaC*<br>10 mM EDTA	39	48	7.2 x 10^{-14}	183	-7.5	4B
MG1655<br>100 mM EGTA	MG1655<br>100 mM EDTA	35	31	1.8 x 10^{-3}	299	-3.1	4B
OAR<br>100 mM EGTA	OAR<br>100 mM EDTA	32	37	6.7 x 10^{-2}	440	-1.8	4B
Δ*waaC*<br>10 mM EDTA	MG1655<br>100 mM EDTA	48	31	0.07	927	1.8	4B
Δ*waaC*<br>10 mM EDTA	OAR<br>100 mM EDTA	48	37	0.03	639	-2.2	4B
MG1655<br>100 mM EDTA	OAR<br>100 mM EDTA	31	37	0.09	711	1.7	4B
Δ*waaC*<br>0.025% (w/v) LDAO	MG1655<br>0.025% (w/v) LDAO	30	37	0.64	503	-0.5	4B
Δ*waaC*<br>0.025% (w/v) LDAO	OAR<br>0.025% (w/v) LDAO	30	35	8.1 x 10^{-12}	1045	6.8	4B
MG1655<br>0.025% (w/v) LDAO	OAR<br>0.025% (w/v) LDAO	37	35	1.3 x 10^{-11}	1219	6.8	4B
Δ*waaC*<br>2.0 mM Mg^{2+}, 0.1 mM Ca^{2+}	Δ*waaC*<br>0.1 mM Mg^{2+}, 0.1 mM Ca^{2+}	49	39	5.7 x 10^{-9}	261	-5.8	5B
Δ*waaC*<br>2.0 mM Mg^{2+}, 0.1 mM Ca^{2+}	Δ*waaC*<br>0.1 mM Mg^{2+}, 2.0 mM Ca^{2+}	49	51	1.8 x 10^{-10}	324	-6.4	5B
MG1655<br>2.0 mM Mg^{2+}, 0.1 mM Ca^{2+}	MG1655<br>0.1 mM Mg^{2+}, 0.1 mM Ca^{2+}	74	30	4.8 x 10^{-7}	408	-5.0	5B
MG1655<br>2.0 mM Mg^{2+}, 0.1 mM Ca^{2+}	MG1655<br>0.1 mM Mg^{2+}, 2.0 mM Ca^{2+}	74	30	9.0 x 10^{-11}	206	-6.5	5B
OAR<br>2.0 mM Mg^{2+}, 0.1 mM Ca^{2+}	OAR<br>0.1 mM Mg^{2+}, 0.1 mM Ca^{2+}	74	39	1.8 x 10^{-8}	520	-5.6	5B
OAR<br>2.0 mM Mg^{2+}, 0.1 mM Ca^{2+}	OAR<br>0.1 mM Mg^{2+}, 2.0 mM Ca^{2+}	74	32	1.8 x 10^{-12}	163	-7.1	5B
OAR<br>No treatment	OAR<br>50 mM EGTA, 50 mM EDTA	74	31	1.9 x 10^{-5}	547	-4.3	7C
E. coli UTI89<br>No treatment	*E. coli* UTI89<br>50 mM EGTA, 50 mM EDTA	31	38	8.4 x 10^{-5}	247	-3.9	7C
S. Typhimurium LT2<br>No treatment	*S.* Typhimurium LT2<br>50 mM EGTA, 50 mM EDTA	33	43	2.8 x 10^{-9}	151	-5.9	7C
P. aeruginosa PAO1<br>No treatment	*P. aeruginosa* PAO1<br>50 mM EGTA, 50 mM EDTA	30	30	3.0 x 10^{-7}	103	-5.1	7C
OAR<br>No treatment	*E. coli* UTI89<br>No treatment	74	32	0.051	781	-2.9	7C
OAR<br>No treatment	*S.* Typhimurium LT2<br>No treatment	74	33	7.8 x 10^{-4}	772	-3.4	7C
OAR<br>No treatment	*P. aeruginosa* PAO1<br>No treatment	74	30	6.0 x 10^{-3}	811	-2.7	7C
CuAAC<br>Δ*waaC*	SPAAC<br>Δ*waaC*	84	32	0.11	1085	-1.6	EV2B
imp4213<br>No treatment	*imp4213*<br>100 mM EGTA	49	31	0.10	606	-1.6	EV4B
imp4213<br>No treatment	*imp4213*<br>100 mM EDTA	49	35	0.75	912	0.3	EV4B
imp4213<br>No treatment	MG1655<br>100 mM EDTA	49	31	0.36	871	0.9	EV4B
MG1655<br>No treatment	MG1655<br>300 mM Urea	74	30	8.2 x 10^{-2}	741	-2.6	S9A
OAR<br>No treatment	OAR<br>300 mM Urea	74	35	0.20	1128	-1.3	S9A
UTI89<br>No treatment	UTI89<br>300 mM Urea	31	35	4.1 x 10^{-4}	267	-3.5	S9A
MG1655	ClearColi™	74	58	7.15 x 10^{-17}	340	-8.3	6C
MG1655<br>0.025% (w/v) LDAO	ClearColi™	37	58	0.0070	1450	2.70	
Δ*waaC*<br>0.025% (w/v) LDAO	ClearColi™	30	58	0.0078	578	-2.66	

Appendix Table S2: Cell numbers (n) for FRAP data and median mobile fraction values for cells cultured in M9 CDM with modified divalent cation concentrations (no chelator data plotted in Figure 5)

Strain	M9 CDM type	Treatment	n	Median mobile fraction
Δ*waaC*	high Ca^{2+}, low Mg^{2+}	none	51	0.15
Δ*waaC*	high Ca^{2+}, low Mg^{2+}	5 mM EDTA, 5 mM EGTA	39	0.21
Δ*waaC*	low Ca^{2+} low Mg^{2+}	none	44	0.15
Δ*waaC*	low Ca^{2+} low Mg^{2+}	5 mM EDTA, 5 mM EGTA	35	0.19
Δ*waaC*	low Ca^{2+} high Mg^{2+}	none	49	0.09
Δ*waaC*	low Ca^{2+} high Mg^{2+}	5 mM EDTA, 5 mM EGTA	30	0.24
MG1655	high Ca^{2+}, low Mg^{2+}	none	30	0.18
MG1655	high Ca^{2+}, low Mg^{2+}	50 mM EDTA, 50 mM EGTA	58	0.23
MG1655	low Ca^{2+} low Mg^{2+}	none	34	0.18
MG1655	low Ca^{2+} low Mg^{2+}	50 mM EDTA, 50 mM EGTA	31	0.17
MG1655	low Ca^{2+} high Mg^{2+}	none	74	0.09
MG1655	low Ca^{2+} high Mg^{2+}	50 mM EDTA, 50 mM EGTA	31	0.18
OAR	high Ca^{2+}, low Mg^{2+}	none	32	0.20
OAR	high Ca^{2+}, low Mg^{2+}	50 mM EDTA, 50 mM EGTA	42	0.20
OAR	low Ca^{2+} low Mg^{2+}	none	39	0.15
OAR	low Ca^{2+} low Mg^{2+}	50 mM EDTA, 50 mM EGTA	30	0.16
OAR	low Ca^{2+} high Mg^{2+}	none	75	0.08
OAR	low Ca^{2+} high Mg^{2+}	50 mM EDTA, 50 mM EGTA	31	0.17

Appendix Table S3: Cell numbers (n) for FRAP data and median mobile fraction values for bacterial cells with and without combined EDTA and EGTA treatment (data plotted in Figure 7)

**Strain**	**Treatment**	**n**	**Median mobile fraction**
E. coli OAR	none	75	0.08
	50 mM EDTA, 50 mM EGTA	31	0.17
E. coli UTI89	none	44	0.12
	50 mM EDTA, 50 mM EGTA	35	0.15
S. Typhimurium LT2	none	49	0.11
	50 mM EDTA, 50 mM EGTA	30	0.19
P. aeruginosa PAO1	none	30	0.11
	50 mM EDTA, 50 mM EGTA	58	0.18

Appendix Table S4. Details of Gram-negative bacterial strains used in this study

Strain	Organism	LPS oligosaccharide length†	Selection antibiotic	Notes
**K-12 substr. MG1655**	*E. coli*	Rough	None	Contains *rfb-50* mutation that results in absence of O-antigen synthesis.
**Δ*waaC***	*E. coli*	Deep rough	30 μg mL^{-1} KAN	Keio collection strain JW3596-KC. *ΔwaaC 733*::Kan prohibits extension of LPS oligosachride domain beyond Kdo residues.
**ClearColi™**	*E. coli*	Endotoxin-free	None	Endotoxin-free strain (MG1655 background) with Lipid IV_A as the only LPS-related glycolipid in the OM. This strain was only grown in LB-Miller medium.
**BW25113 GFP-TolA**	*E. coli*	Rough	30 μg mL^{-1} KAN	BW25113 strain transformed with pNP4 plasmid to produce GFP-tagged TolA as described previously.
**Δ*lpp***	*E. coli*	Rough	30 μg mL^{-1} KAN	Keio collection strain JW1667-KC.
imp4213	*E. coli*	Rough	None	BW25113 background with *lptD* in-frame deletion (D330 – D352) mutation resulting in defective LptD function.
imp4213* pBAD-lptD	*E. coli*	Rough	100 μg mL^{-1} AMP 35 μg mL^{-1} CHL	*imp4213* transformed with pBAD-lptD* pEVOL-pylRS plasmids. Produces recombinant LptD with ncAA Lys analogue at position D600 when provided with the ncAA.
**Δ*ompA* pBAD-ompA***	*E. coli*	Rough	100 μg mL^{-1} AMP 35 μg mL^{-1} CHL 30 μg mL^{-1} KAN	Keio collection strain JW0940-KC transformed with pBAD-ompA* pEVOL-pylRS plasmid. Produces recombinant OmpA with ncAA Lys analogue at position E89 when provided with the ncAA.
**DFB1655 OAR**	*E. coli*	Smooth	30 μg mL^{-1} KAN	K-12 substr. MG1655 with *wbbL* integrated downstream of IS5 element. Presents O16 serotype O-antigen.
**LT2**	*Salmonella enterica enterica* serovar Typhimurium strain LT2	Smooth	None	Pathogenic model strain, serotype I 4,5,12:i:1,2.
**PAO1**	*Pseudomonas auerginosa*	Smooth	None	Opportunistic pathogen model bacterial strain isolated from a wound in a patient. Capsule-forming serotype.
**UTI89**	*E. coli*	Smooth	None	Uropathogenic *E. coli* strain (UPEC) O18:K1:H7. Capsule-forming serotype.

† LPS oligosaccharide type classifications: Deep rough = Lipid A-$(Kdo)_2$, **Rough =** Lipid A-$(Kdo)_2$ plus core oligosachrides, **Smooth =** Lipid A-$(Kdo)_2$ plus core oligosachrides and 1 – 40 penta-saccharide O-antigen repeat units. **Antibiotic abbreviations: KAN =** Kanamycin (Sigma-Aldrich), **AMP =** Ampicillin (Melford Laboratories Ltd.), **CHL =** Chloramphenicol (Sigma-Aldrich)

Appendix Table S5. DNA primer sequences

Primer ID	Sequence (5' → 3')	Notes
lptD^{WT}-for	TTTGGGCTAACAGGAGGAATTACATATGAAAAAACGTATCCCCACTCTCC	*lptD* gene PCR amplification
lptD^{WT}-rev	GAGATGAGTTTTTGTTCTAGAAAGCTTACAAAGTGTTTTGATACGGCAGA	*lptD* gene PCR amplification
pBADcLIC-for	TCTGCCGTATCAAAACACTTTGTAAGCTTTCTAGAACAAAAACTCATCTC	pBADcLIC vector PCR amplification
pBADcLIC-rev	GGAGAGTGGGGATACGTTTTTTCATATGTAATTCCTCCTGTTAGCCCAAA	pBADcLIC vector PCR amplification
pBADcLIC_forS	ATGCCATAGCATTTTTATCC	pBAD-lptD sequencing
pBADcLIC-revS	GATTTAATCTGTATCAGG	pBAD-lptD sequencing
lptDmid_forS	TACTTTGAGTTCTACCTGCC	pBAD-lptD sequencing
lptDmid_revS	ATGCTGGAGTTACTGGTCGC	pBAD-lptD sequencing
lptD$^{[D600]}$-forM	CGATGACAACATAACATGGGAGAATTAGGACAAAACGGGTTCACTGGT	pBAD-lptD PCR mutagenesis
lptD$^{[D600]}$-revM	ACCAGTGAACCCGTTTTGTCCTAATTCTCCCATGTTATGTTGTCATCG	pBAD-lptD PCR mutagensis
ompA^{WT}-for	GCTAACAGGAGGAATTAACCATGGATGAAAAAGACAGCTATCGCGATTG	*ompA* gene PCR amplification
ompA^{WT}-rev	GATGAGTTTTTGTTCTAGAAAGCTTCGTTAAGCCTGCGGCTGAGTTAC	*ompA* gene PCR amplification
pBAD-for-2	CGTTGTAACTCAGCCGCAGGCTTAACGAAGCTTTCTAGAACAAAAACTCATCTCAG	pBADcLIC vector PCR amplification
pBAD-rev-2	CAATCGCGATAGCTGTCTTTTTCATCCATGGTTAATTCCTCCTGTTAGCC	pBADcLIC vector PCR amplification
ompA$^{[E89]}$-for	CGTATGCCGTACAAAGGCAGCGTTTAGAACGGTGCATACAAAGCTCAGGGC	pBAD-ompA PCR mutagenesis
ompA$^{[E89]}$-rev	GCCCTGAGCTTTGTATGCACCGTTCTAAACGCTGCCTTTGTACGGCATACG	pBAD-ompA PCR mutagenesis

Appendix Table S6. Composition of supplemented M9 chemically defined medium (pH 7.2)

Component	Concentration
Na_2HPO_4	48.0 mM
KH_2PO_4	22.0 mM
NaCl	08.6 mM
D-glucose	0.4% (w/v)
NH_4Cl	1.0 g L^{-1}
Casamino acids	0.05% (w/v)
$FeSO_4$	0.1 mM
$MgSO_4$	2.0 mM
$CaCl_2$	0.1 mM

Appendix Table S7: Final concentrations of alkyne functionalized dyes used in copper-catalyzed CuAAC reaction mixtures

Dye type	**Final concentration** (µM)
AF488 or AZDye 488	10
AZDye 568	20
AZDye 594	10
AZ647	50

Appendix Table S8. Final concentrations of AZDye-DBCO dyes (Vector Laboratories, Inc.) used in copper-free SPAAC reaction mixtures

AZDye DBCO type	**Concentration** (µM)
AZDye 488	25
AZDye 568	50

Appendix Table S9. Propargyl-OmpA^{E89} / Kdo-N_3-LPS dual labeling combinations used to ensure fluorescent dye type and labeling order did not influence two-color dSTORM results

Experimental replicate	First labeled species / fluorescent dye	Second labeled species / fluorescent dye
1	LPS / AZ488-alkyne	OmpA^{E89} / AZ647-N_3
2	OmpA^{E89} / AZ647-N_3	LPS / AZ488-alkyne
3	LPS / AZ647-alkyne	OmpA^{E89} / AZ488-N_3
4	OmpA^{E89} / AZ488-N_3	LPS / AZ647-alkyne

Appendix Table S10. 4x TSDS-PAGE sample loading buffer composition

Component	Final concentration
Tris-Cl pH 6.8	0.25 M
Sodium dodecyl sulfate (SDS)	227 mM
Bromophenol blue	0.02 % (w/v)
Glycerol	4.3 M
**Note:** β-ME added to a final concentration 2% (v/v) immediately prior to its usage.	

Appendix Table S11. Concentrations of chelator, detergent and urea used for pre-FRAP treatments

LPS oligosaccharide glycoform	**EGTA conc.** (mM)	**EDTA conc.** (mM)	**EGTA / EDTA conc.** (mM)	**LDAO conc.** (%, w/v)	**Urea conc.** (mM)
Deep rough	10	10	5 / 5	0.025	300
Rough	100	100	50 / 50	0.025	300
Smooth	100	100	50 / 50	0.025	300

Appendix Table S12. Composition of supplemented M9 chemically defined medium (CDM), supplemented M9 CDM with reduced [Mg^{2+}] and supplemented M9 CDM with elevated [Ca^{2+}] (differences across the three media types are highlighted in bold).

Component	**Standard M9 CDM pH 7.2 (high [Mg^{2+}])**	**Low [Mg^{2+}] M9 CDM pH 7.2**	**High [Ca^{2+}] M9 CDM pH 7.2**
NaCl	0.5 g L^{-1}	0.5 g L^{-1}	0.5 g L^{-1}
Na_2HPO_4	6.78 g L^{-1}	6.78 g L^{-1}	6.78 g L^{-1}
KH_2PO_4	3.0 g L^{-1}	3.0 g L^{-1}	3.0 g L^{-1}
NH_4Cl	1.0 g L^{-1}	1.0 g L^{-1}	1.0 g L^{-1}
casamino acids	0.05% (w/v)	0.05% (w/v)	0.05% (w/v)
D-glucose	22 mM	22 mM	22 mM
**$MgSO_4$**	2 mM	**100 µM**	100 µM
**$CaCl_2$**	100 µM	100 µM	**2 mM**
$FeSO_4$	100 µM	100 µM	100 µM

Appendix Table S13. Upright Zeiss LSM710 confocal microscope settings

Dye	MBS	Laser	Emission filter (nm)	Laser power (%)	Pinhole size (µm)	Master gain	Bleach iterations
AF488	488	488 nm Argon	BP 493 – 630	3 – 7	53.3	350 – 400	20
DIC for AF488	488 / 561	561 nm DPSS	N/A	2	53.3	250 – 300	N/A
AF568	488/561	561 nm DPSS	BP 568 – 712	2 – 5	90.0	500 – 600	30
DIC for AF568	488	488 nm Argon	N/A	2	90.0	250 – 300	N/A
AZ647	488/561/633	633 DPSS	BP 638 – 756	2 – 5	90.0	600 – 700	10
DIC for AZ647	488	488 nm Argon	N/A	2	90.0	250 – 300	N/A

DIC: Differential interference contrast. **MBS**: Confocal main beam splitter dichromatic mirror. **BP**: Band pass. **DPSS:** Diode pumped solid state. Images collected using a Plan-Apochromat 63x/1.4 (DIC M27) oil immersion objective lens. Pixel dwell time: 3.15 µs. Image size: 512 pixels x 512 pixels (px), collected as 16-bit images. Scan time: 3.87 s. For FRAP photobleaching regions were 50 px by 30 px orientated at one end of the target cell. FRAP image series were collected with 10x digital zoom, photobleaching laser power set at 100%. DIC images for labeled samples were collected using a laser that emitted at a different wavelength to the λ_{ex} of the fluorescent dye label thus minimizing photobleaching.

Appendix Table S14. Inverted Zeiss LSM780 confocal multiphoton microscope settings

Dye	MBS	Laser	Emission filter (nm)	Laser power (%)	Pinhole size (µm)	Master gain	Bleach iterations
AF488	488 / 561	488 nm Argon	BP 493 – 630	2 – 3	47.7	700 – 750	20
DIC for AF488	488 / 561	561 nm DPSS	N/A	2	53.3	250 – 300	N/A
AF568	488 / 561	561 nm DPSS	BP 568 – 712	2 – 5	90.0	400 – 500	30
DIC for AF568	488	488 nm Argon	N/A	2	90.0	250 – 300	N/A

DIC: Differential interference contrast. **MBS**: Confocal main beam splitter dichromatic mirror. **BP**: Band pass. **HeNe:** Helium-neon. Images collected using a Plan-Apochromat 63x/1.4 (DIC M27) oil immersion objective lens. Pixel dwell time: 1.58 µs. Image size: 512 pixels x 512 pixels, collected as 16-bit images. Scan time: 1.94 s.

Appendix Table S15. Components in Glucose oxidase (GluOx) / Catalase (Cat) oxygen scavenging buffer system composition

**Component**	**Volume** (µL)
Degassed deionized H_2O	895
PBS pH 7.4 [40 x] (BioStatus)	25
GluOx [10 mg mL^{-1}]	20
Cat [2 mg mL^{-1}]	20
Glucose [300 mg mL^{-1}]	40

Appendix Table S16. Additional settings for Zeiss Elyra 7 SRM microscope 2D dSTORM image acquisition

**Dye**	**Laser beam splitter**	**Laser**	**Emission filter** (nm)	**xy scaling** (µm pixel^{-1})	**TIRF mirror angle**	**TIRF collimator**
AZ488	405/488/561/642	488 nm DPSS	BP420-480 + BP490-550	0.097	60°	230
AZ647	405/488/561/642	561 nm DPSS	BP570-620 + LP655	0.097	60°	400

Appendix Table S17. Typical dSTORM image filtering settings

**Filter**	**AF488 dye**	**AZ647 dye**
Precision (nm)	1 – 60	1 – 60
Number of photons	1 – 2000	1 – 1250
Point squared function half width (nm)	60 – 200	100 – 300
Background variance	1 – 300	1 – 300
Chi-squared	0.4 – 1.2	0.4 – 1.2
