# Supplementary figures and images for: Lipopolysaccharide confinement in the bacterial outer membrane is governed by interactions within the conserved Lipid A anchor (part 1 of 2)
Source: EMBO J. 2026 Feb 17;45(7):2338–69. doi: 10.1038/s44318-026-00711-5 (PMC13043748; doi:10.1038/s44318-026-00711-5)

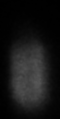

Supplement: Supplementary file 4 — Source data Fig. 1 [file 44318_2026_711_MOESM4_ESM.zip › EMBOJ-2025-121562-Figure-1-source-data/1B/1B AF594-PG/AF594-PG-1-min-post-bleach.tif]

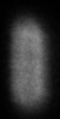

Supplement: Supplementary file 4 — Source data Fig. 1 [file 44318_2026_711_MOESM4_ESM.zip › EMBOJ-2025-121562-Figure-1-source-data/1B/1B AF594-PG/AF594-PG-initial-bleach-sequence.tif]

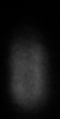

Supplement: Supplementary file 4 — Source data Fig. 1 [file 44318_2026_711_MOESM4_ESM.zip › EMBOJ-2025-121562-Figure-1-source-data/1B/1B AF594-PG/AF594-PG-5-min-post-bleach.tif]

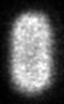

Supplement: Supplementary file 4 — Source data Fig. 1 [file 44318_2026_711_MOESM4_ESM.zip › EMBOJ-2025-121562-Figure-1-source-data/1B/1B GFP-TolA/GFP-TolA-5-min-post-bleach.tif]

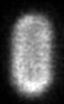

Supplement: Supplementary file 4 — Source data Fig. 1 [file 44318_2026_711_MOESM4_ESM.zip › EMBOJ-2025-121562-Figure-1-source-data/1B/1B GFP-TolA/GFP-TolA-1-min-post-bleach.tif]

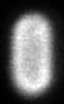

Supplement: Supplementary file 4 — Source data Fig. 1 [file 44318_2026_711_MOESM4_ESM.zip › EMBOJ-2025-121562-Figure-1-source-data/1B/1B GFP-TolA/GFP-TolA-initial-bleach-sequence.tif]

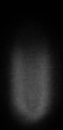

Supplement: Supplementary file 4 — Source data Fig. 1 [file 44318_2026_711_MOESM4_ESM.zip › EMBOJ-2025-121562-Figure-1-source-data/1B/1B AF488-LPS/AF488-LPS-5-min-post-bleach.tif]

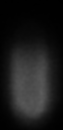

Supplement: Supplementary file 4 — Source data Fig. 1 [file 44318_2026_711_MOESM4_ESM.zip › EMBOJ-2025-121562-Figure-1-source-data/1B/1B AF488-LPS/AF488-LPS-1-min-post-bleach.tif]

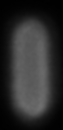

Supplement: Supplementary file 4 — Source data Fig. 1 [file 44318_2026_711_MOESM4_ESM.zip › EMBOJ-2025-121562-Figure-1-source-data/1B/1B AF488-LPS/AF488-LPS-initial-bleach-sequence.tif]

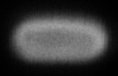

Supplement: Supplementary file 4 — Source data Fig. 1 [file 44318_2026_711_MOESM4_ESM.zip › EMBOJ-2025-121562-Figure-1-source-data/1E/1E-AF488-LPS-delta-waaC/AF488-LPS-delta-waaC-initial-bleach-sequence.tif]

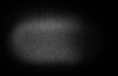

Supplement: Supplementary file 4 — Source data Fig. 1 [file 44318_2026_711_MOESM4_ESM.zip › EMBOJ-2025-121562-Figure-1-source-data/1E/1E-AF488-LPS-delta-waaC/AF488-LPS-delta-waaC-5-min-post-bleach.tif]

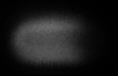

Supplement: Supplementary file 4 — Source data Fig. 1 [file 44318_2026_711_MOESM4_ESM.zip › EMBOJ-2025-121562-Figure-1-source-data/1E/1E-AF488-LPS-delta-waaC/AF488-LPS-delta-waaC-1-min-post-bleach.tif]

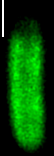

Supplement: Supplementary file 4 — Source data Fig. 1 [file 44318_2026_711_MOESM4_ESM.zip › EMBOJ-2025-121562-Figure-1-source-data/1E/1E-AF488-LPS-OAR/AF488-LPS-OAR-5-min-post-bleach.tif]

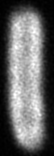

Supplement: Supplementary file 4 — Source data Fig. 1 [file 44318_2026_711_MOESM4_ESM.zip › EMBOJ-2025-121562-Figure-1-source-data/1E/1E-AF488-LPS-OAR/AF488-LPS-OAR-initial-bleach-sequence.tif]

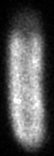

Supplement: Supplementary file 4 — Source data Fig. 1 [file 44318_2026_711_MOESM4_ESM.zip › EMBOJ-2025-121562-Figure-1-source-data/1E/1E-AF488-LPS-OAR/AF488-LPS-OAR-1-min-post-bleach.tif]

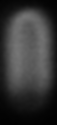

Supplement: Supplementary file 7 — Source data Fig. 4 [file 44318_2026_711_MOESM7_ESM.zip › EMBOJ-2025-121562-Figure-4-source-data/4A/4A delta-waaC EGTA/delta-waaC-EGTA-1-min-post-bleach.tif]

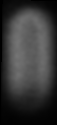

Supplement: Supplementary file 7 — Source data Fig. 4 [file 44318_2026_711_MOESM7_ESM.zip › EMBOJ-2025-121562-Figure-4-source-data/4A/4A delta-waaC EGTA/delta-waaC-EGTA-5-min-post-bleach.tif]

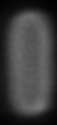

Supplement: Supplementary file 7 — Source data Fig. 4 [file 44318_2026_711_MOESM7_ESM.zip › EMBOJ-2025-121562-Figure-4-source-data/4A/4A delta-waaC EGTA/delta-waaC-EGTA-initial-bleach-sequence.tif]

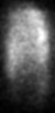

Supplement: Supplementary file 7 — Source data Fig. 4 [file 44318_2026_711_MOESM7_ESM.zip › EMBOJ-2025-121562-Figure-4-source-data/4A/4A delta-waaC LDAO/delta-waaC-LDAO-1-min-post-bleach.tif]

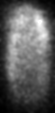

Supplement: Supplementary file 7 — Source data Fig. 4 [file 44318_2026_711_MOESM7_ESM.zip › EMBOJ-2025-121562-Figure-4-source-data/4A/4A delta-waaC LDAO/delta-waaC-LDAO-5-min-post-bleach.tif]

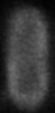

Supplement: Supplementary file 7 — Source data Fig. 4 [file 44318_2026_711_MOESM7_ESM.zip › EMBOJ-2025-121562-Figure-4-source-data/4A/4A delta-waaC LDAO/delta-waaC-LDAO-initial-bleach-sequence.tif]

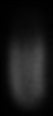

Supplement: Supplementary file 7 — Source data Fig. 4 [file 44318_2026_711_MOESM7_ESM.zip › EMBOJ-2025-121562-Figure-4-source-data/4A/4A MG1655 EGTA/MG1655-EGTA-1-min-post-bleach.tif]

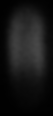

Supplement: Supplementary file 7 — Source data Fig. 4 [file 44318_2026_711_MOESM7_ESM.zip › EMBOJ-2025-121562-Figure-4-source-data/4A/4A MG1655 EGTA/MG1655-EGTA-5-min-post-bleach.tif]

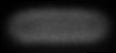

Supplement: Supplementary file 7 — Source data Fig. 4 [file 44318_2026_711_MOESM7_ESM.zip › EMBOJ-2025-121562-Figure-4-source-data/4A/4A MG1655 EGTA/MG1655-EGTA-initial-bleach-sequence.tif]

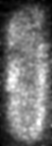

Supplement: Supplementary file 7 — Source data Fig. 4 [file 44318_2026_711_MOESM7_ESM.zip › EMBOJ-2025-121562-Figure-4-source-data/4A/4A MG1655 LDAO/MG1655-LDAO-1-min-post-bleach.tif]

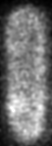

Supplement: Supplementary file 7 — Source data Fig. 4 [file 44318_2026_711_MOESM7_ESM.zip › EMBOJ-2025-121562-Figure-4-source-data/4A/4A MG1655 LDAO/MG1655-LDAO-5-min-post-bleach.tif]

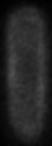

Supplement: Supplementary file 7 — Source data Fig. 4 [file 44318_2026_711_MOESM7_ESM.zip › EMBOJ-2025-121562-Figure-4-source-data/4A/4A MG1655 LDAO/MG1655-LDAO-initial-bleach-sequence.tif]

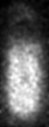

Supplement: Supplementary file 7 — Source data Fig. 4 [file 44318_2026_711_MOESM7_ESM.zip › EMBOJ-2025-121562-Figure-4-source-data/4A/4A OAR EDTA/OAR-EDTA-1-min-post-bleach.tif]

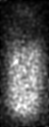

Supplement: Supplementary file 7 — Source data Fig. 4 [file 44318_2026_711_MOESM7_ESM.zip › EMBOJ-2025-121562-Figure-4-source-data/4A/4A OAR EDTA/OAR-EDTA-5-min-post-bleach.tif]

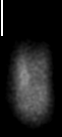

Supplement: Supplementary file 7 — Source data Fig. 4 [file 44318_2026_711_MOESM7_ESM.zip › EMBOJ-2025-121562-Figure-4-source-data/4A/4A OAR EGTA/OAR-EGTA-1-min-post-bleach.tif]

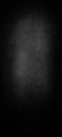

Supplement: Supplementary file 7 — Source data Fig. 4 [file 44318_2026_711_MOESM7_ESM.zip › EMBOJ-2025-121562-Figure-4-source-data/4A/4A OAR EGTA/OAR-EGTA-5-min-post-bleach.tif]

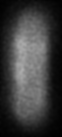

Supplement: Supplementary file 7 — Source data Fig. 4 [file 44318_2026_711_MOESM7_ESM.zip › EMBOJ-2025-121562-Figure-4-source-data/4A/4A OAR EGTA/OAR-EGTA-initial-bleach-sequence.tif]

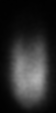

Supplement: Supplementary file 7 — Source data Fig. 4 [file 44318_2026_711_MOESM7_ESM.zip › EMBOJ-2025-121562-Figure-4-source-data/4A/4A OAR LDAO/OAR-LDAO-1-min-post-bleach.tif]

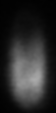

Supplement: Supplementary file 7 — Source data Fig. 4 [file 44318_2026_711_MOESM7_ESM.zip › EMBOJ-2025-121562-Figure-4-source-data/4A/4A OAR LDAO/OAR-LDAO-5-min-post-bleach.tif]

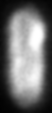

Supplement: Supplementary file 7 — Source data Fig. 4 [file 44318_2026_711_MOESM7_ESM.zip › EMBOJ-2025-121562-Figure-4-source-data/4A/4A OAR LDAO/OAR-LDAO-initial-bleach-sequence.tif]

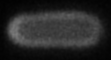

Supplement: Supplementary file 8 — Source data Fig. 5 [file 44318_2026_711_MOESM8_ESM.zip › EMBOJ-2025-121562-Figure-5-source-data/5A/delta-waaC-high-Ca-low-Mg/delta-waaC-high-Ca-low-Mg-initial-bleach-sequence.tif]

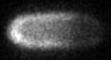

Supplement: Supplementary file 8 — Source data Fig. 5 [file 44318_2026_711_MOESM8_ESM.zip › EMBOJ-2025-121562-Figure-5-source-data/5A/delta-waaC-high-Ca-low-Mg/delta-waaC-high-Ca-low-Mg-1-min-post-bleach.tif]

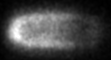

Supplement: Supplementary file 8 — Source data Fig. 5 [file 44318_2026_711_MOESM8_ESM.zip › EMBOJ-2025-121562-Figure-5-source-data/5A/delta-waaC-high-Ca-low-Mg/delta-waaC-high-Ca-low-Mg-5-min-post-bleach.tif]

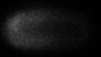

Supplement: Supplementary file 8 — Source data Fig. 5 [file 44318_2026_711_MOESM8_ESM.zip › EMBOJ-2025-121562-Figure-5-source-data/5A/MG1655-high-Ca-low-Mg/MG1655-high-Ca-low-Mg-1-min-post-bleach.tif]

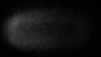

Supplement: Supplementary file 8 — Source data Fig. 5 [file 44318_2026_711_MOESM8_ESM.zip › EMBOJ-2025-121562-Figure-5-source-data/5A/MG1655-high-Ca-low-Mg/MG1655-high-Ca-low-Mg-5-min-post-bleach.tif]

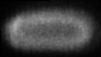

Supplement: Supplementary file 8 — Source data Fig. 5 [file 44318_2026_711_MOESM8_ESM.zip › EMBOJ-2025-121562-Figure-5-source-data/5A/MG1655-high-Ca-low-Mg/MG1655-high-Ca-low-Mg-initial-bleach-sequence.tif]

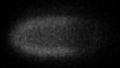

Supplement: Supplementary file 8 — Source data Fig. 5 [file 44318_2026_711_MOESM8_ESM.zip › EMBOJ-2025-121562-Figure-5-source-data/5A/delta-waaC-low-Ca-low-Mg/delta-waaC-low-Ca-low-Mg-1-min-post-bleach.tif]

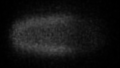

Supplement: Supplementary file 8 — Source data Fig. 5 [file 44318_2026_711_MOESM8_ESM.zip › EMBOJ-2025-121562-Figure-5-source-data/5A/delta-waaC-low-Ca-low-Mg/delta-waaC-low-Ca-low-Mg-5-min-post-bleach.tif]

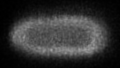

Supplement: Supplementary file 8 — Source data Fig. 5 [file 44318_2026_711_MOESM8_ESM.zip › EMBOJ-2025-121562-Figure-5-source-data/5A/delta-waaC-low-Ca-low-Mg/delta-waaC-low-Ca-low-Mg-initial-bleach-sequence.tif]

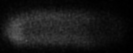

Supplement: Supplementary file 8 — Source data Fig. 5 [file 44318_2026_711_MOESM8_ESM.zip › EMBOJ-2025-121562-Figure-5-source-data/5A/MG1655-low-Ca-low-Mg/MG1655-low-Ca-low-Mg-1-min-post-bleach.tif]

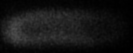

Supplement: Supplementary file 8 — Source data Fig. 5 [file 44318_2026_711_MOESM8_ESM.zip › EMBOJ-2025-121562-Figure-5-source-data/5A/MG1655-low-Ca-low-Mg/MG1655-low-Ca-low-Mg-5-min-post-bleach.tif]

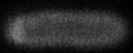

Supplement: Supplementary file 8 — Source data Fig. 5 [file 44318_2026_711_MOESM8_ESM.zip › EMBOJ-2025-121562-Figure-5-source-data/5A/MG1655-low-Ca-low-Mg/MG1655-low-Ca-low-Mg-initial-bleach-sequence.tif]

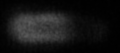

Supplement: Supplementary file 8 — Source data Fig. 5 [file 44318_2026_711_MOESM8_ESM.zip › EMBOJ-2025-121562-Figure-5-source-data/5A/OAR-high-Ca-low-Mg/OAR-high-Ca-low-Mg-1-min-post-bleach.tif]

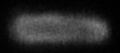

Supplement: Supplementary file 8 — Source data Fig. 5 [file 44318_2026_711_MOESM8_ESM.zip › EMBOJ-2025-121562-Figure-5-source-data/5A/OAR-high-Ca-low-Mg/OAR-high-Ca-low-Mg-initial-bleach-sequence.tif]

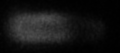

Supplement: Supplementary file 8 — Source data Fig. 5 [file 44318_2026_711_MOESM8_ESM.zip › EMBOJ-2025-121562-Figure-5-source-data/5A/OAR-high-Ca-low-Mg/OAR-high-Ca-low-Mg-5-min-post-bleach.tif]

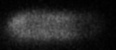

Supplement: Supplementary file 8 — Source data Fig. 5 [file 44318_2026_711_MOESM8_ESM.zip › EMBOJ-2025-121562-Figure-5-source-data/5A/OAR-low-Ca-low-Mg/OAR-low-Ca-low-Mg-5-miin-post-bleach.tif]

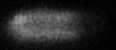

Supplement: Supplementary file 8 — Source data Fig. 5 [file 44318_2026_711_MOESM8_ESM.zip › EMBOJ-2025-121562-Figure-5-source-data/5A/OAR-low-Ca-low-Mg/OAR-low-Ca-low-Mg-1-miin-post-bleach.tif]

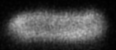

Supplement: Supplementary file 8 — Source data Fig. 5 [file 44318_2026_711_MOESM8_ESM.zip › EMBOJ-2025-121562-Figure-5-source-data/5A/OAR-low-Ca-low-Mg/OAR-low-Ca-low-Mg-initial-bleach-sequence.tif]

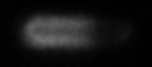

Supplement: Supplementary file 9 — Source data Fig. 6 [file 44318_2026_711_MOESM9_ESM.zip › EMBOJ-2025-121562-Figure-6-source-data/6B/ClearColi/ClearColi-1-min-post-bleach.tif]

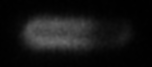

Supplement: Supplementary file 9 — Source data Fig. 6 [file 44318_2026_711_MOESM9_ESM.zip › EMBOJ-2025-121562-Figure-6-source-data/6B/ClearColi/ClearColi-5-min-post-bleach.tif]

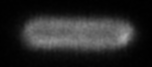

Supplement: Supplementary file 9 — Source data Fig. 6 [file 44318_2026_711_MOESM9_ESM.zip › EMBOJ-2025-121562-Figure-6-source-data/6B/ClearColi/ClearColi-initial-bleach-sequence.tif]

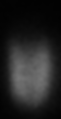

Supplement: Supplementary file 9 — Source data Fig. 6 [file 44318_2026_711_MOESM9_ESM.zip › EMBOJ-2025-121562-Figure-6-source-data/6B/MG1655/MG1655-1-min-post-bleach.tif]

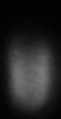

Supplement: Supplementary file 9 — Source data Fig. 6 [file 44318_2026_711_MOESM9_ESM.zip › EMBOJ-2025-121562-Figure-6-source-data/6B/MG1655/MG1655-5-min-post-bleach.tif]

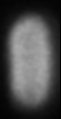

Supplement: Supplementary file 9 — Source data Fig. 6 [file 44318_2026_711_MOESM9_ESM.zip › EMBOJ-2025-121562-Figure-6-source-data/6B/MG1655/MG1655-initial-bleach-sequence.tif]

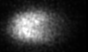

Supplement: Supplementary file 10 — Source data Fig. 7 [file 44318_2026_711_MOESM10_ESM.zip › EMBOJ-2025-121562-Figure-7-source-data/7B/7B PAO1 no treatment/PAO1-no-treatment-1-min-post-bleach.tif]

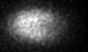

Supplement: Supplementary file 10 — Source data Fig. 7 [file 44318_2026_711_MOESM10_ESM.zip › EMBOJ-2025-121562-Figure-7-source-data/7B/7B PAO1 no treatment/PAO1-no-treatment-5-min-post-bleach.tif]

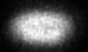

Supplement: Supplementary file 10 — Source data Fig. 7 [file 44318_2026_711_MOESM10_ESM.zip › EMBOJ-2025-121562-Figure-7-source-data/7B/7B PAO1 no treatment/PAO1-no-treatment-initial-bleach-sequence.tif]

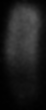

Supplement: Supplementary file 10 — Source data Fig. 7 [file 44318_2026_711_MOESM10_ESM.zip › EMBOJ-2025-121562-Figure-7-source-data/7B/7B UTI89 EDTA EGTA/UTI89-EDTA-EGTA-5-min-post-bleach.tif]

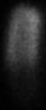

Supplement: Supplementary file 10 — Source data Fig. 7 [file 44318_2026_711_MOESM10_ESM.zip › EMBOJ-2025-121562-Figure-7-source-data/7B/7B UTI89 EDTA EGTA/UTI89-EDTA-EGTA-1-min-post-bleach.tif]

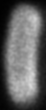

Supplement: Supplementary file 10 — Source data Fig. 7 [file 44318_2026_711_MOESM10_ESM.zip › EMBOJ-2025-121562-Figure-7-source-data/7B/7B UTI89 EDTA EGTA/UTI89-EDTA-EGTA-initial-bleach-sequence.tif]

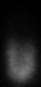

Supplement: Supplementary file 10 — Source data Fig. 7 [file 44318_2026_711_MOESM10_ESM.zip › EMBOJ-2025-121562-Figure-7-source-data/7B/7B LT2 no treatment/LT2-no-treatment-1-min-post-bleach.tif]

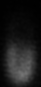

Supplement: Supplementary file 10 — Source data Fig. 7 [file 44318_2026_711_MOESM10_ESM.zip › EMBOJ-2025-121562-Figure-7-source-data/7B/7B LT2 no treatment/LT2-no-treatment-5-min-post-bleach.tif]

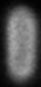

Supplement: Supplementary file 10 — Source data Fig. 7 [file 44318_2026_711_MOESM10_ESM.zip › EMBOJ-2025-121562-Figure-7-source-data/7B/7B LT2 no treatment/LT2-no-treatment-initial-bleach-sequence.tif]

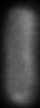

Supplement: Supplementary file 10 — Source data Fig. 7 [file 44318_2026_711_MOESM10_ESM.zip › EMBOJ-2025-121562-Figure-7-source-data/7B/7B LT2 EDTA EGTA/LT2-EDTA-EGTA-initial-bleach-sequence.tif]

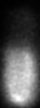

Supplement: Supplementary file 10 — Source data Fig. 7 [file 44318_2026_711_MOESM10_ESM.zip › EMBOJ-2025-121562-Figure-7-source-data/7B/7B LT2 EDTA EGTA/LT2-EDTA-EGTA-5-min-post-bleach.tif]

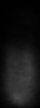

Supplement: Supplementary file 10 — Source data Fig. 7 [file 44318_2026_711_MOESM10_ESM.zip › EMBOJ-2025-121562-Figure-7-source-data/7B/7B LT2 EDTA EGTA/LT2-EDTA-EGTA-1-min-post-bleach.tif]

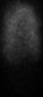

Supplement: Supplementary file 10 — Source data Fig. 7 [file 44318_2026_711_MOESM10_ESM.zip › EMBOJ-2025-121562-Figure-7-source-data/7B/7B UTI89 no treatment/UTI89-no-treatment-1-min-post-bleach.tif]

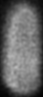

Supplement: Supplementary file 10 — Source data Fig. 7 [file 44318_2026_711_MOESM10_ESM.zip › EMBOJ-2025-121562-Figure-7-source-data/7B/7B UTI89 no treatment/UTI89-no-treatment-initial-bleach-sequence.tif]

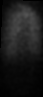

Supplement: Supplementary file 10 — Source data Fig. 7 [file 44318_2026_711_MOESM10_ESM.zip › EMBOJ-2025-121562-Figure-7-source-data/7B/7B UTI89 no treatment/UTI89-no-treatment-5-min-post-bleach.tif]

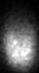

Supplement: Supplementary file 10 — Source data Fig. 7 [file 44318_2026_711_MOESM10_ESM.zip › EMBOJ-2025-121562-Figure-7-source-data/7B/7B PAO1 EDTA EGTA/PAO1-EDTA-EGTA-1-min-post-bleach.tif]

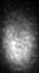

Supplement: Supplementary file 10 — Source data Fig. 7 [file 44318_2026_711_MOESM10_ESM.zip › EMBOJ-2025-121562-Figure-7-source-data/7B/7B PAO1 EDTA EGTA/PAO1-EDTA-EGTA-5-min-post-bleach.tif]

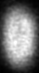

Supplement: Supplementary file 10 — Source data Fig. 7 [file 44318_2026_711_MOESM10_ESM.zip › EMBOJ-2025-121562-Figure-7-source-data/7B/7B PAO1 EDTA EGTA/PAO1-EDTA-EGTA-initial-bleach-sequence.tif]

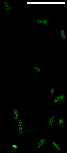

Supplement: Supplementary file 11 — Appendix Figure Source Data [file 44318_2026_711_MOESM11_ESM.zip › Appendix Figure S1/S1A/S1A-delta-waaC-AF488-LPS.tif]

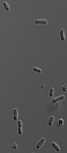

Supplement: Supplementary file 11 — Appendix Figure Source Data [file 44318_2026_711_MOESM11_ESM.zip › Appendix Figure S1/S1A/S1A-delta-waaC-DIC.tif]

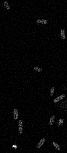

Supplement: Supplementary file 11 — Appendix Figure Source Data [file 44318_2026_711_MOESM11_ESM.zip › Appendix Figure S1/S1A/S1A-delta-waaC-AF488-LPS-DIC-composite.tif]

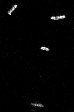

Supplement: Supplementary file 11 — Appendix Figure Source Data [file 44318_2026_711_MOESM11_ESM.zip › Appendix Figure S1/S1C/S1C-OAR-AF488-LPS-DIC-composite.tif]

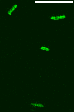

Supplement: Supplementary file 11 — Appendix Figure Source Data [file 44318_2026_711_MOESM11_ESM.zip › Appendix Figure S1/S1C/S1C-OAR-AF488-LPS.tif]

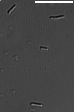

Supplement: Supplementary file 11 — Appendix Figure Source Data [file 44318_2026_711_MOESM11_ESM.zip › Appendix Figure S1/S1C/S1C-OAR-DIC.tif]

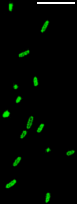

Supplement: Supplementary file 11 — Appendix Figure Source Data [file 44318_2026_711_MOESM11_ESM.zip › Appendix Figure S1/S1B/S1B-MG1655-AF488-LPS.tif]

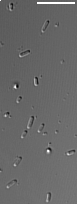

Supplement: Supplementary file 11 — Appendix Figure Source Data [file 44318_2026_711_MOESM11_ESM.zip › Appendix Figure S1/S1B/S1B-MG1655-DIC.tif]

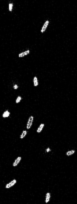

Supplement: Supplementary file 11 — Appendix Figure Source Data [file 44318_2026_711_MOESM11_ESM.zip › Appendix Figure S1/S1B/S1B-MG1655-AF488-LPS-DIC-composite.tif]

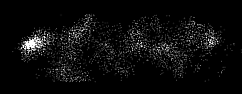

Supplement: Supplementary file 11 — Appendix Figure Source Data [file 44318_2026_711_MOESM11_ESM.zip › Appendix Figure S2/S2B-AF488-OmpA-AZ647-LPS-dSTORM-composite.tif]

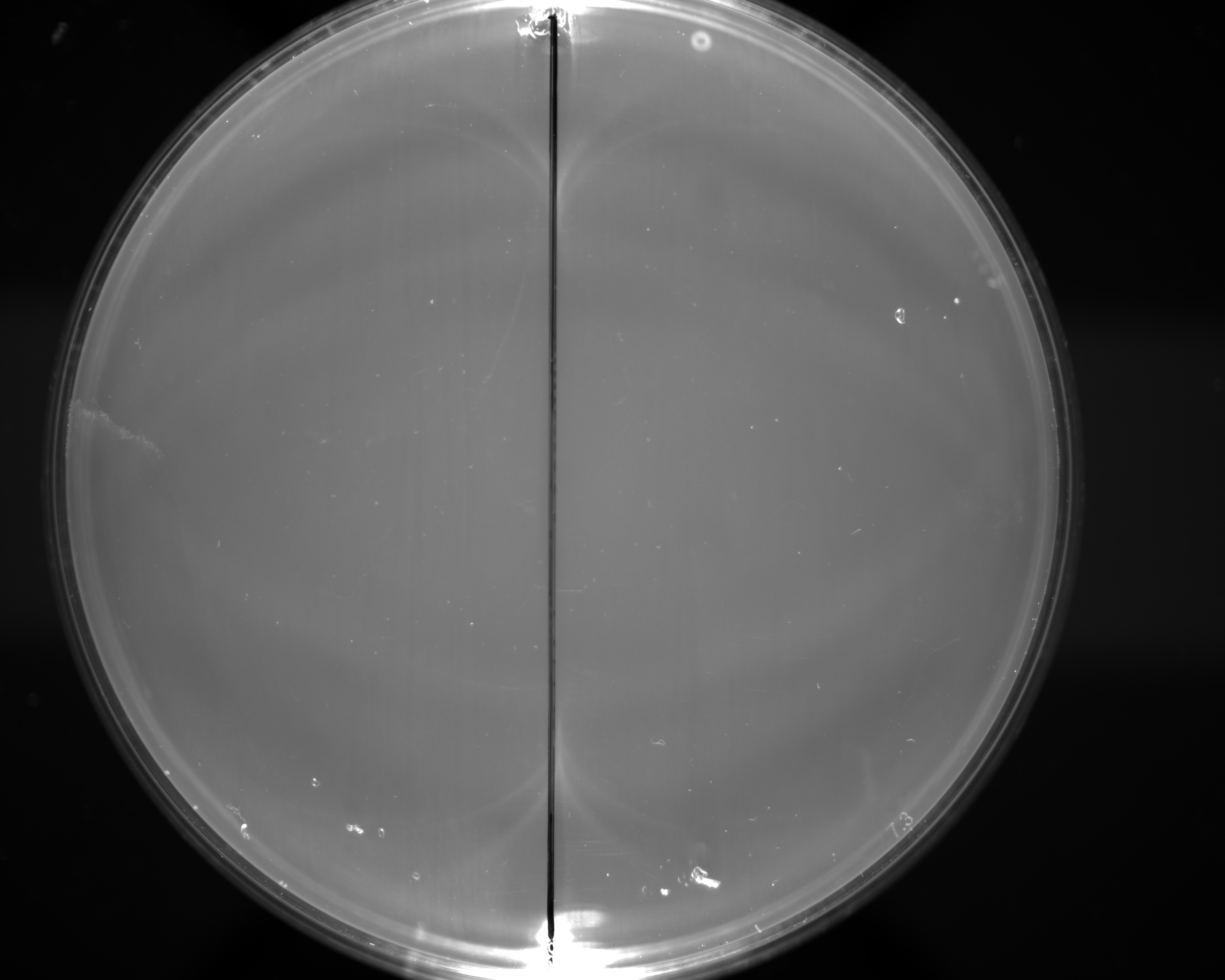

Supplement: Supplementary file 11 — Appendix Figure Source Data [file 44318_2026_711_MOESM11_ESM.zip › Appendix Figure S3/Figure-S3-without-ncaa-top-right-agar-plate.tif]

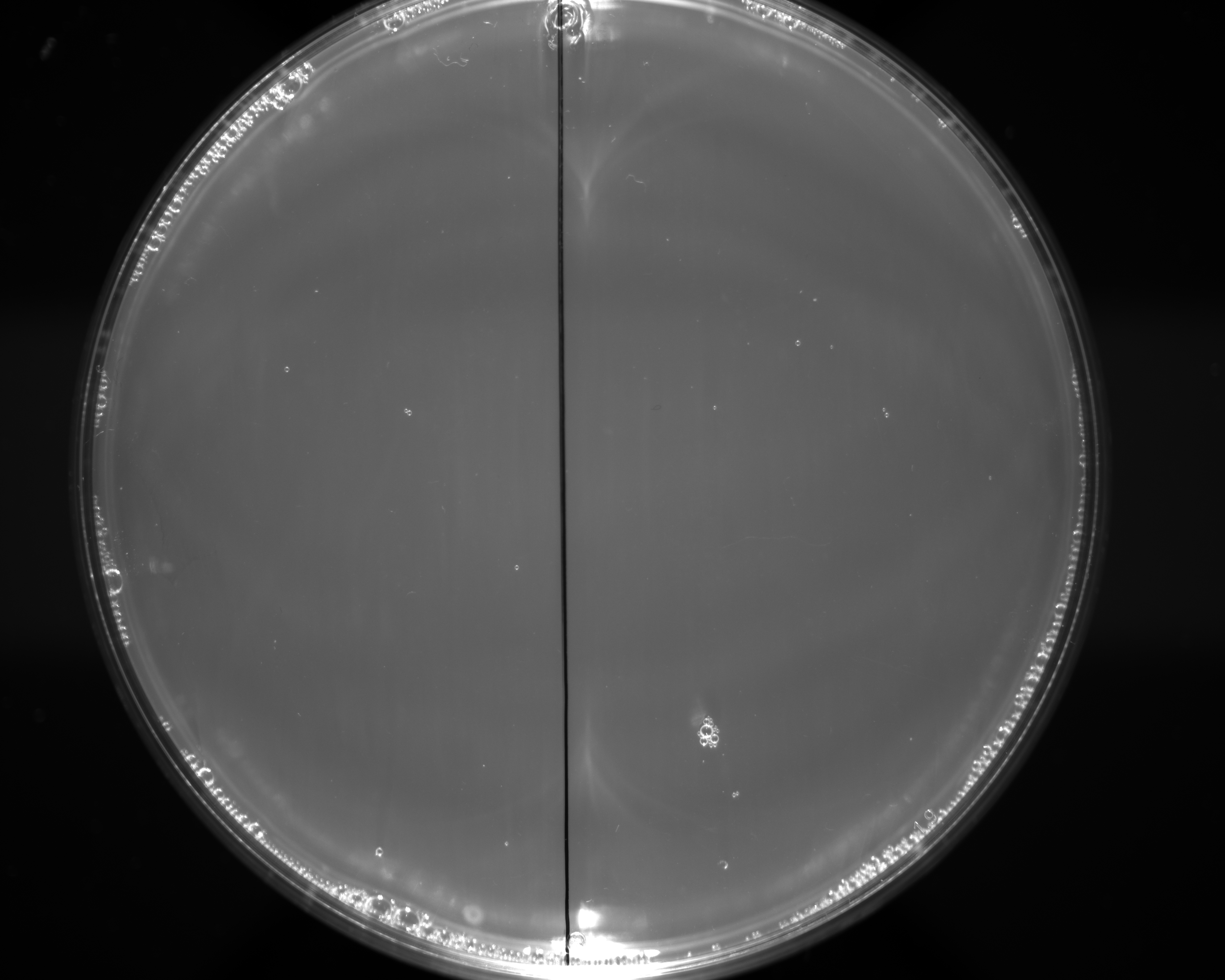

Supplement: Supplementary file 11 — Appendix Figure Source Data [file 44318_2026_711_MOESM11_ESM.zip › Appendix Figure S3/Figure-S3-without-ncaa-top-left-agar-plate.tif]

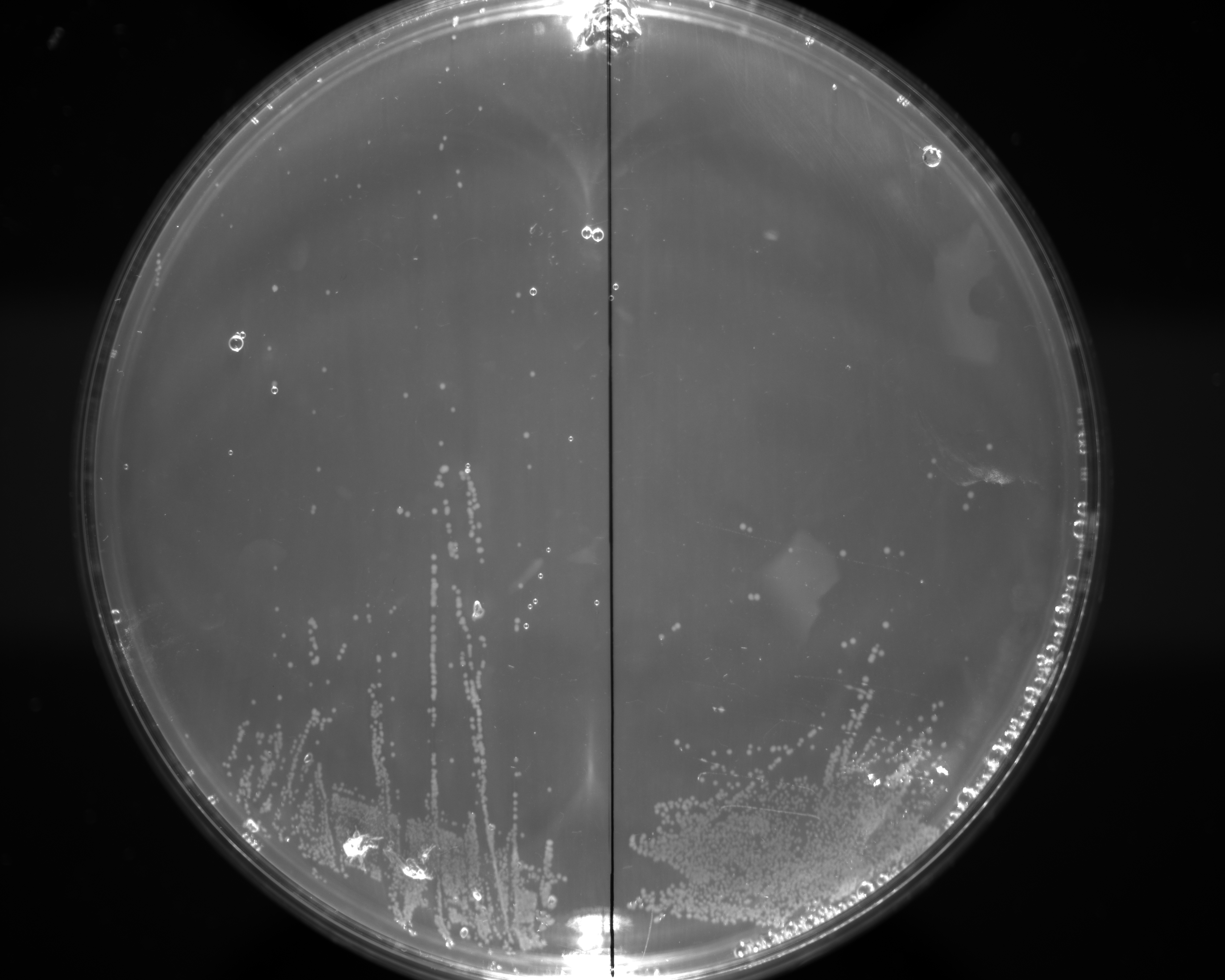

Supplement: Supplementary file 11 — Appendix Figure Source Data [file 44318_2026_711_MOESM11_ESM.zip › Appendix Figure S3/Figure-S3-with-ncaa-bottom-right-agar-plate.tif]

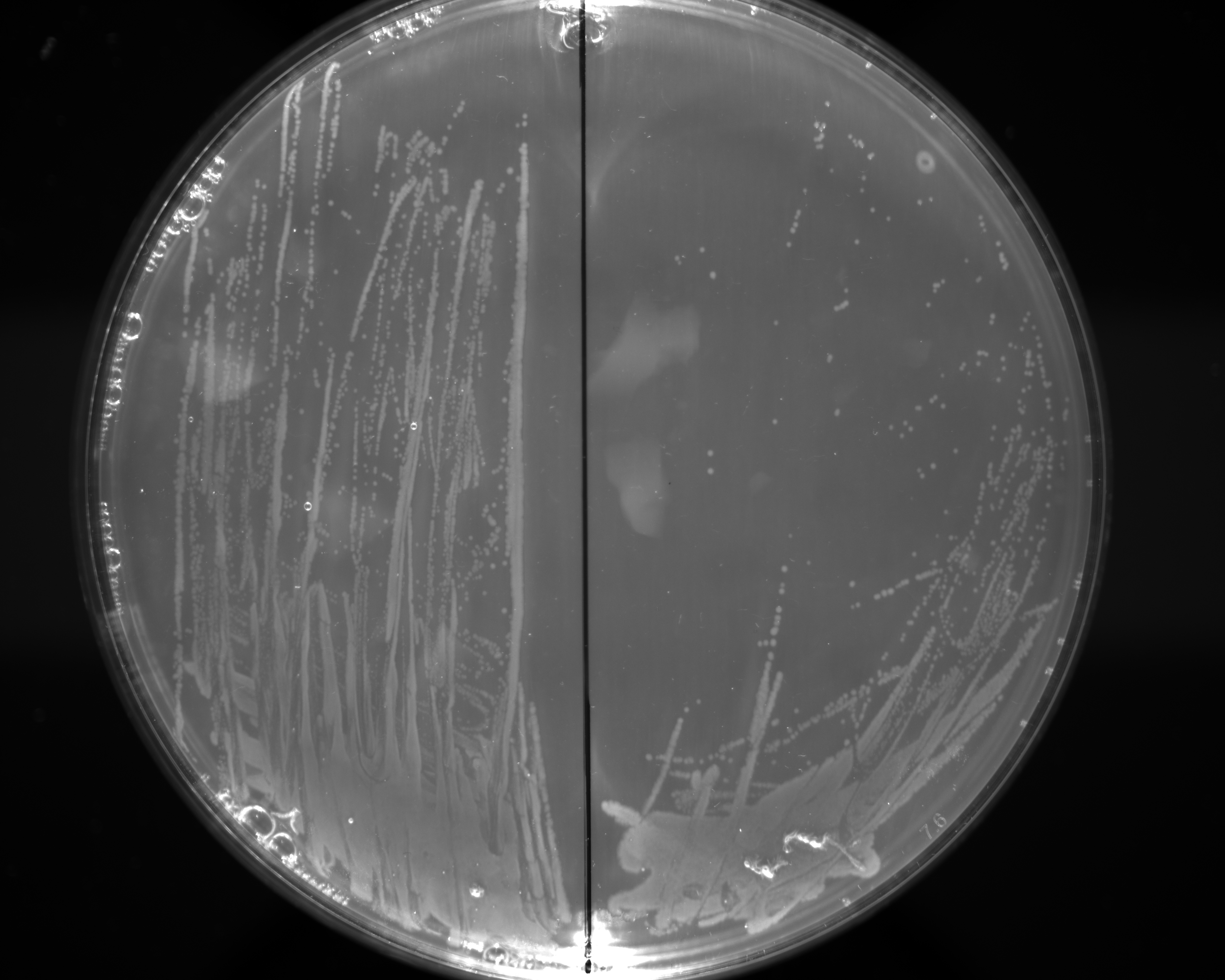

Supplement: Supplementary file 11 — Appendix Figure Source Data [file 44318_2026_711_MOESM11_ESM.zip › Appendix Figure S3/Figure-S3-with-ncaa-bottom-left-agar-plate.tif]

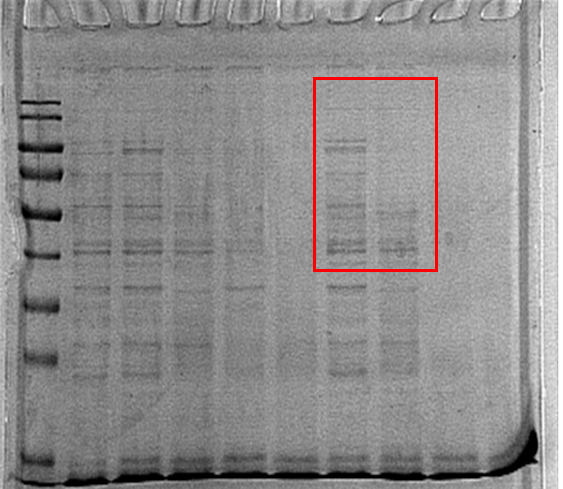

Supplement: Supplementary file 11 — Appendix Figure Source Data [file 44318_2026_711_MOESM11_ESM.zip › Appendix Figure S4/S4B/S4B-TSDS-PAGE-gel-Coomassie-stained.tif]

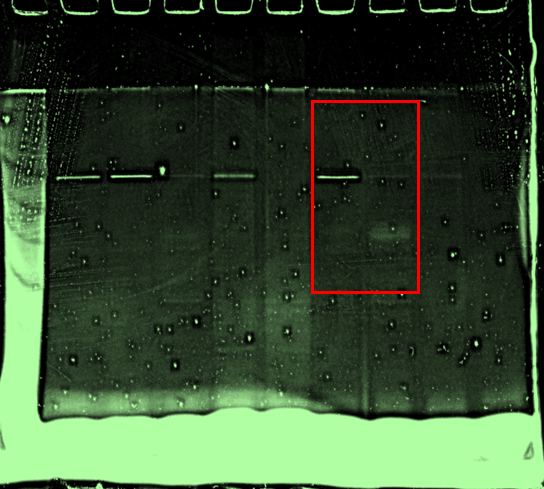

Supplement: Supplementary file 11 — Appendix Figure Source Data [file 44318_2026_711_MOESM11_ESM.zip › Appendix Figure S4/S4B/S4B-TSDS-PAGE-gel-AZ488-fluorescence.tif]

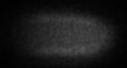

Supplement: Supplementary file 12 — Figure EV2 Source Data [file 44318_2026_711_MOESM12_ESM.zip › Expanded View Figure 2/EV2A/delta waaC SPAAC AF568-LPS/delta-waaC-SPAAC-AF568-1-min-post-bleach.tif]

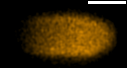

Supplement: Supplementary file 12 — Figure EV2 Source Data [file 44318_2026_711_MOESM12_ESM.zip › Expanded View Figure 2/EV2A/delta waaC SPAAC AF568-LPS/delta-waaC-SPAAC-AF568-5-min-post-bleach.tif]

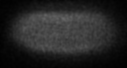

Supplement: Supplementary file 12 — Figure EV2 Source Data [file 44318_2026_711_MOESM12_ESM.zip › Expanded View Figure 2/EV2A/delta waaC SPAAC AF568-LPS/delta-waaC-SPAAC-AF568-initial-bleach-sequence.tif]

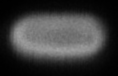

Supplement: Supplementary file 12 — Figure EV2 Source Data [file 44318_2026_711_MOESM12_ESM.zip › Expanded View Figure 2/EV2A/delta waaC CuAAC AF488-LPS/delta-waaC-CuAAC-AF488-initial-bleach-sequence.tif]

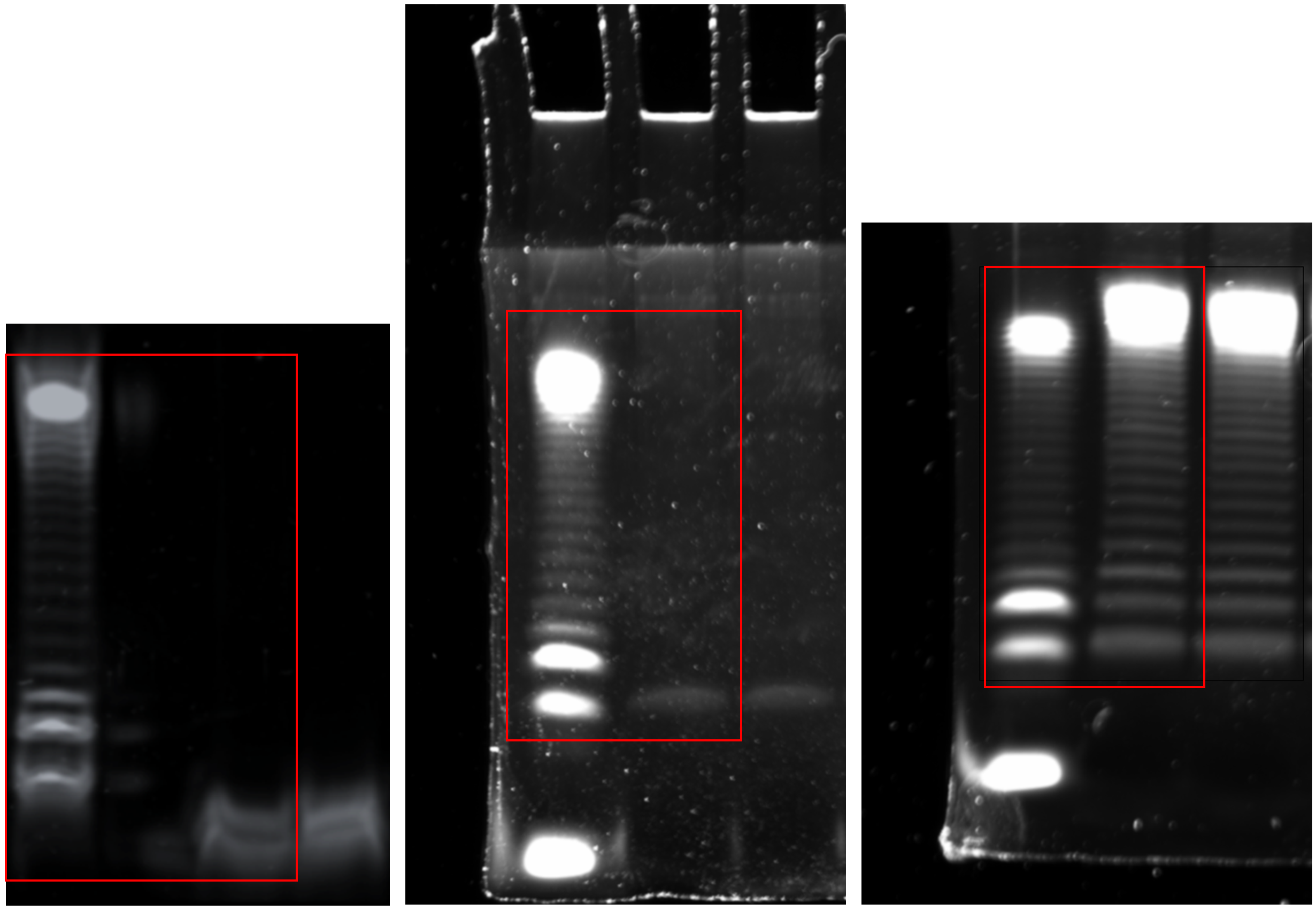

Supplement: Supplementary file 13 — Figure EV3 Source Data [file 44318_2026_711_MOESM13_ESM.zip › Expanded View Figure 3/Figure-EV3-TSDS-PAGE-bottom-three-panels.tif]

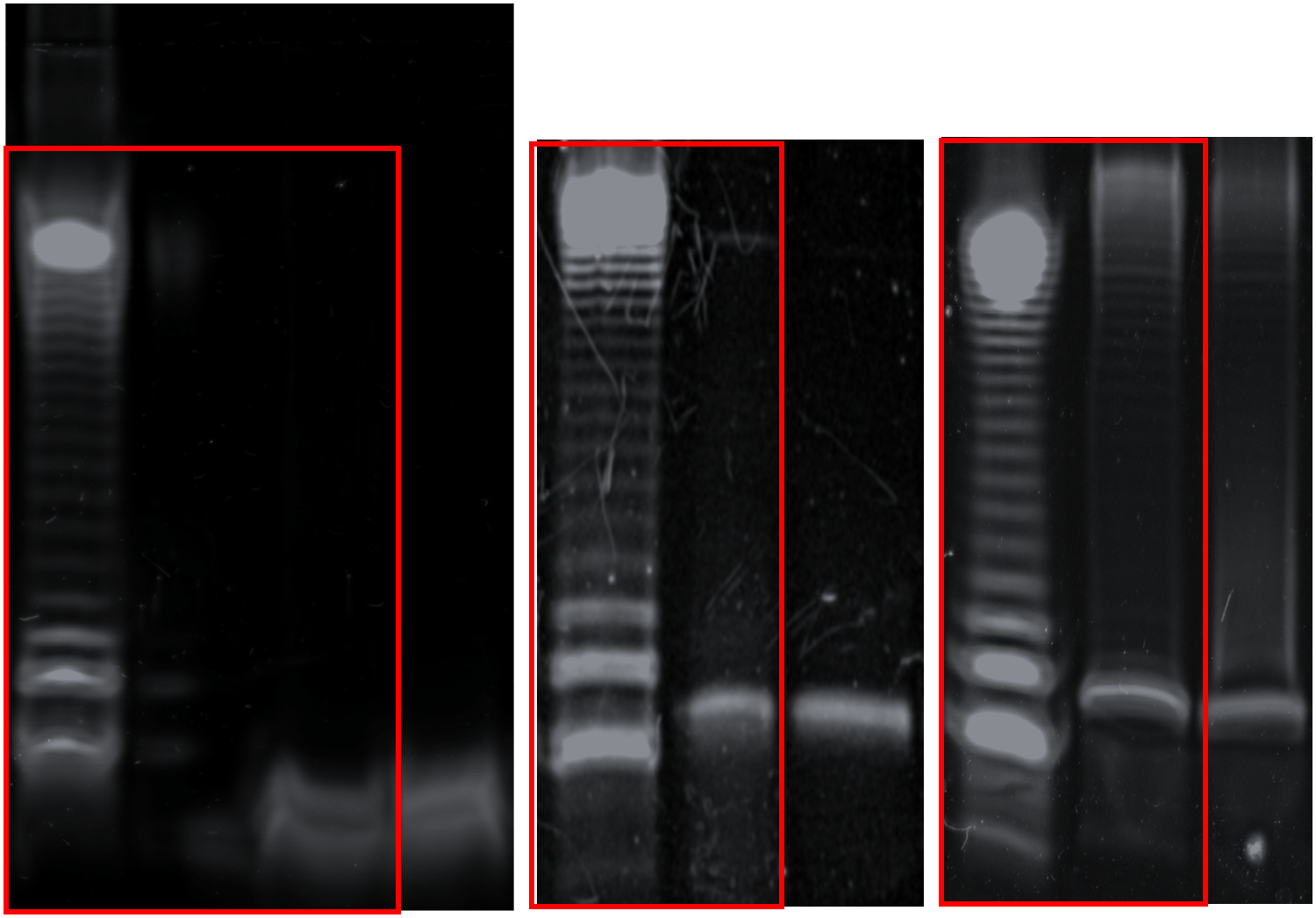

Supplement: Supplementary file 13 — Figure EV3 Source Data [file 44318_2026_711_MOESM13_ESM.zip › Expanded View Figure 3/Figure-EV3-TSDS-PAGE-top-three-panels.tif]

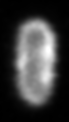

Supplement: Supplementary file 14 — Figure EV4 Source Data [file 44318_2026_711_MOESM14_ESM.zip › Expanded View Figure 4/EV4A/EV4A imp4213 no treatment/imp4213-no-treatment-initial-bleach-sequence.tif]
