# Supplementary material for: Lipopolysaccharide confinement in the bacterial outer membrane is governed by interactions within the conserved Lipid A anchor
Source: EMBO J. 2026 Feb 17;45(7):2338–69. doi: 10.1038/s44318-026-00711-5 (PMC13043748; doi:10.1038/s44318-026-00711-5)
Supplement: Supplementary file 5 — Source data Fig. 2 [file 44318_2026_711_MOESM5_ESM.zip › 2A/2A-dSTORM-README.docx]

**Figure 2A**

**AZ488-LPS and AZ647-OmpA* two-color dSTORM images**

| 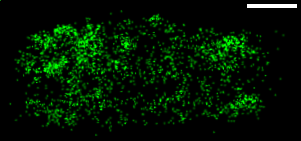 | 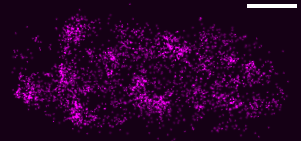 | 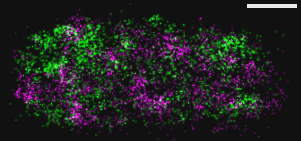 |
| --- | --- | --- |
| **LPS islands (AZ488-LPS)** | **OMP islands (AZ647-OmpA*)** | **Composite** |

**Associated files:**

1. 2A-dSTORM-SMLM-data.xlsx
2. 2A-AZ488-LPS.czi (multi-dimensional image file in Zeiss proprietary format)
3. 2A-AZ647-OmpA.czi (multi-dimensional image file in Zeiss proprietary format)
