# Supplementary material for: Lipopolysaccharide confinement in the bacterial outer membrane is governed by interactions within the conserved Lipid A anchor
Source: EMBO J. 2026 Feb 17;45(7):2338–69. doi: 10.1038/s44318-026-00711-5 (PMC13043748; doi:10.1038/s44318-026-00711-5)
Supplement: Supplementary file 11 — Appendix Figure Source Data [file 44318_2026_711_MOESM11_ESM.zip › Appendix Figure S2/S2B-and S2C-dSTORM-README.docx]

**Appendix Figure S2B**

**AF488-LPS and AZ647-OmpA* two-color dSTORM images**

**Left image:** AF488-LPS

**Middle image:** AZ647-OmpA*

**Right image:** composite of AF488-LPS and AZ647-OmpA* images


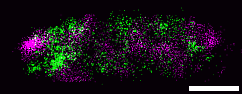

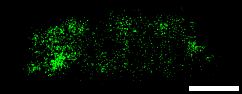

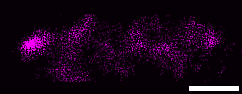


**Associated:**

1. S2B-AF488-OmpA-AZ647-LPS-dSTORM-composite.tif
2. S2B-dSTORM-SMLM-data

**Appendix Figure S2C**

**AF488-OmpA* and AZ647-LPS two-color dSTORM wide-field images**

**Left image:** AZ647-LPS

**Middle image:** AF488-OmpA*


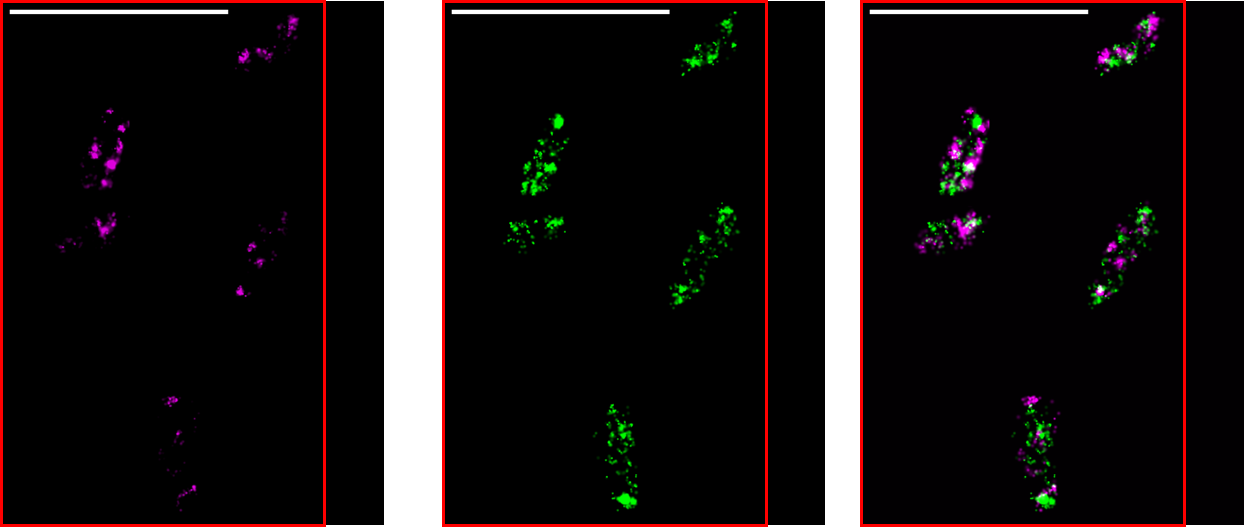
**Right image:** composite of AF488-OmpA* and AZ647-LPS dSTORM wide-field images

**Associated:**

1. S2C-AF488-OmpA.tif
2. S2C-AZ647-LPS.tif
