# Supplementary figures and images for: Lipopolysaccharide confinement in the bacterial outer membrane is governed by interactions within the conserved Lipid A anchor (part 2 of 2)
Source: EMBO J. 2026 Feb 17;45(7):2338–69. doi: 10.1038/s44318-026-00711-5 (PMC13043748; doi:10.1038/s44318-026-00711-5)

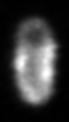

Supplement: Supplementary file 14 — Figure EV4 Source Data [file 44318_2026_711_MOESM14_ESM.zip › Expanded View Figure 4/EV4A/EV4A imp4213 no treatment/imp4213-no-treatment-5-min-post-bleach.tif]

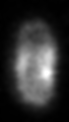

Supplement: Supplementary file 14 — Figure EV4 Source Data [file 44318_2026_711_MOESM14_ESM.zip › Expanded View Figure 4/EV4A/EV4A imp4213 no treatment/imp4213-no-treatment-1-min-post-bleach.tif]

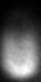

Supplement: Supplementary file 14 — Figure EV4 Source Data [file 44318_2026_711_MOESM14_ESM.zip › Expanded View Figure 4/EV4A/EV4A imp4213 EDTA/imp4213-EDTA-1-min-post-bleach.tif]

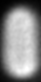

Supplement: Supplementary file 14 — Figure EV4 Source Data [file 44318_2026_711_MOESM14_ESM.zip › Expanded View Figure 4/EV4A/EV4A imp4213 EDTA/imp4213-EDTA-initial-bleach-sequence.tif]

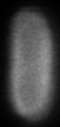

Supplement: Supplementary file 14 — Figure EV4 Source Data [file 44318_2026_711_MOESM14_ESM.zip › Expanded View Figure 4/EV4A/EV4A imp4213 EGTA/imp4213-EGTA-initial-bleach-sequence.tif]

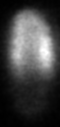

Supplement: Supplementary file 14 — Figure EV4 Source Data [file 44318_2026_711_MOESM14_ESM.zip › Expanded View Figure 4/EV4A/EV4A imp4213 EGTA/imp4213-EGTA-5-min-post-bleach.tif]

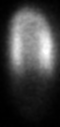

Supplement: Supplementary file 14 — Figure EV4 Source Data [file 44318_2026_711_MOESM14_ESM.zip › Expanded View Figure 4/EV4A/EV4A imp4213 EGTA/imp4213-EGTA-1-min-post-bleach.tif]
