# Supplementary material for: Lipopolysaccharide confinement in the bacterial outer membrane is governed by interactions within the conserved Lipid A anchor
Source: EMBO J. 2026 Feb 17;45(7):2338–69. doi: 10.1038/s44318-026-00711-5 (PMC13043748; doi:10.1038/s44318-026-00711-5)
Supplement: Supplementary file 15 — Expanded View Figures [file 44318_2026_711_MOESM15_ESM.pdf]

Expanded View Figures

Figure EV1. Efficient and specific fluorescent labeling of LPS via a 2-step bio-orthogonal approach enabled LPS lateral mobility to be assessed by in vivo fluorescence microscopy.

(**A**) Fluorescent labeling of LPS at the cell surface. In situ metabolic labeling of the chemically conserved LPS inner core oligosaccharide was done with an azide-functionalized Kdo-analog. Azide handles within LPS inner core oligosaccharide domain enabled bio-orthogonal conjugation of alkyne-functionalized small, photostable organic fluorescence dyes via Cu(I)-catalyzed (CuAAC) or Cu(I)-free strain promoted azide-alkyne cycloaddition (SPAAC). Gal*f* Galactofuranose, Glc Glucose, Rha Rhamnose, GlcNAc *N*-Acetyl-glucosamine, Hep Heptose, Kdo 3-Deoxy-D-manno-oct-2-ulosonic acid, Gal Galactose, GlcN Glucosamine, IM Inner membrane, Lpp Braun's lipoprotein, OMP Outer membrane protein, Und-PP undecaprenyl pyrophosphate anchor. (**B**) Characterization of LPS lateral mobility by fluorescence microscopy. LPS lateral mobility was assessed in a range of Gram-negative bacterial strains under different conditions using (i) fluorescence recovery after photobleaching (FRAP) and (ii) single-particle tracking (SPT) after detection by total internal reflection fluorescence microscopy. The photobleached region of the cell in the schematic for the FRAP experiment has a dashed outline and violet shading. Both confined and free Brownian lateral diffusion could be detected in the SPT experiments, and the type of lateral diffusion observed was found to vary depending on the strain and treatment type.

© The Author(s)

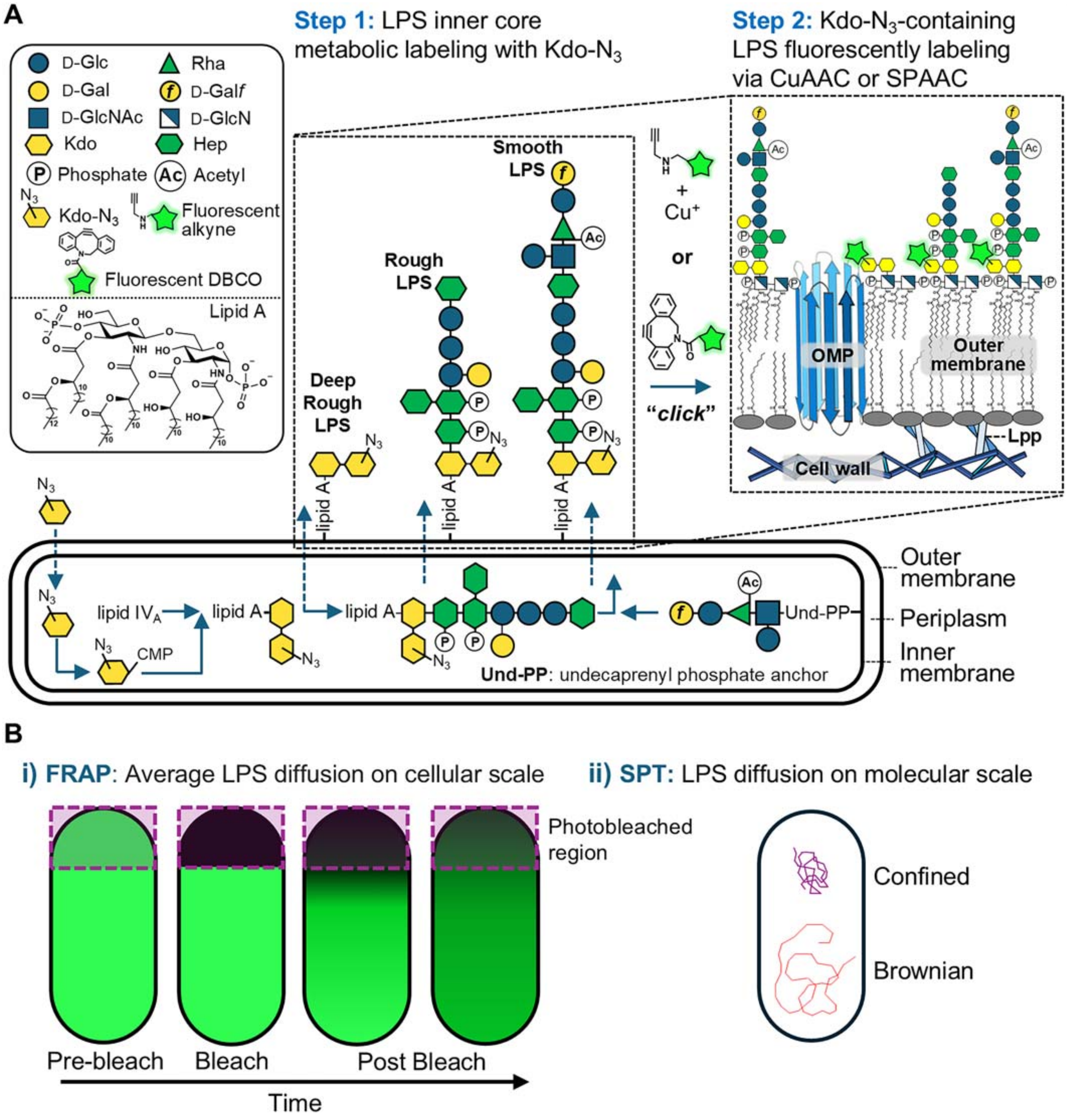
A
Step 1: LPS inner core metabolic labeling with Kdo-N3
Step 2: Kdo-N3-containing LPS fluorescently labeling via CuAAC or SPAAC
D-Glc
Rha
D-Gal
D-Galf
D-GlcNAc
D-GlcN
Kdo
Hep
Phosphate
Acetyl
Kdo-N3
Fluorescent alkyne
Fluorescent DBCO
Lipid A
Smooth LPS
Rough LPS
Deep Rough LPS
Cu+
or
"click"
OMP
Outer membrane
Lpp
Cell wall
lipid IVA
CMP
lipid A
Und-PP
Und-PP: undecaprenyl phosphate anchor
Outer membrane
Periplasm
Inner membrane
B
i) FRAP: Average LPS diffusion on cellular scale
ii) SPT: LPS diffusion on molecular scale
Photobleached region
Pre-bleach
Bleach
Post Bleach
Time
Confined
Brownian

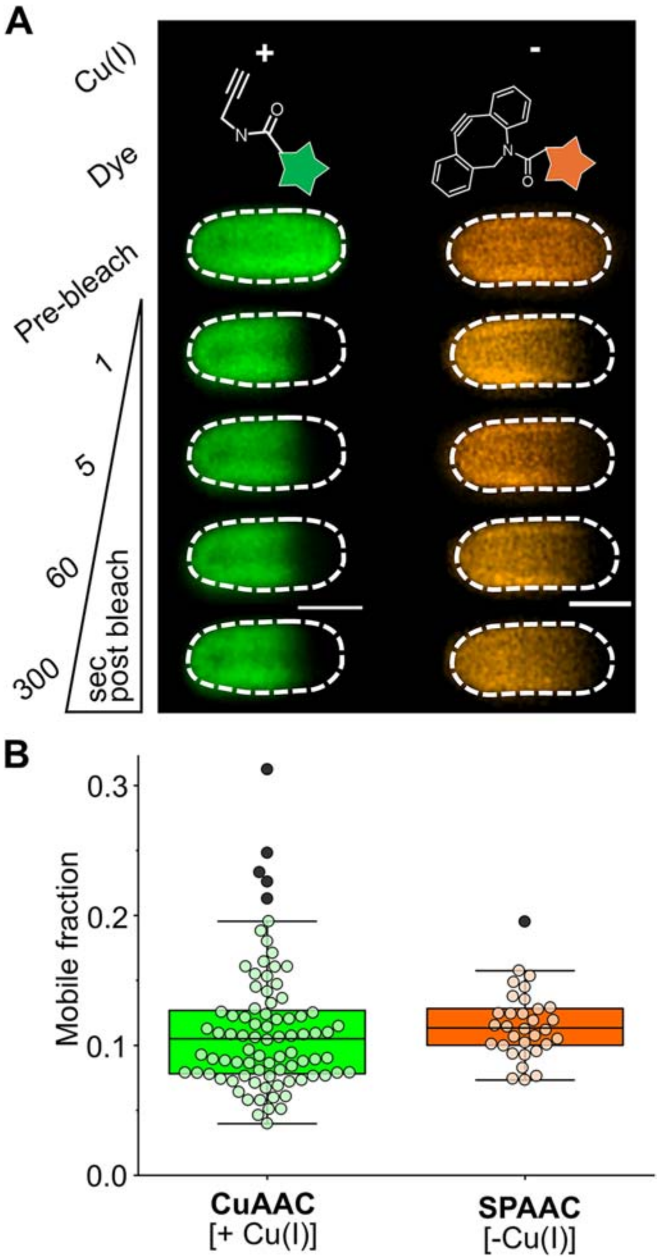

Figure EV2. Exposure to Cu(I) during fluorescent labeling of LPS does not significantly affect LPS lateral mobility as measured by FRAP.

(**A**) Representative FRAP time-lapse images monitoring fluorescence recovery in photobleached regions of the OM for Δ*waaC* cells with LPS labeled using AF488-alkyne via CuAAC (left) or AZDye 568-DBCO via SPAAC (right). Dashed border denotes the outline of each bacterial cell. Scale bars: 1.0 µm. (**B**) No statistically significant difference (by Mann–Whitney test, $P = 0.11$) was observed in the LPS mobile fractions in the OM of *E. coli* Δ*waaC* cells irrespective of whether LPS was labeled via Cu(I)-catalyzed azide-alkyne cycloaddition (CuAAC, green box) or Cu(I)-free strain promoted azide-alkyne cycloaddition (SPAAC, orange box). CuAAC: median mobile fraction = 0.11 ($n = 84$). SPAAC: median mobile fraction = 0.11 ($n = 32$). All FRAP experiments were done in triplicate using independent biological samples. For each box plot, the center line is the median while the box defines the upper and lower quartiles of the data and the whiskers enclose the interquartile range (i.e., middle 50% of the data). Each symbol represents an individual measurement with black-filled symbols classified as outliers (i.e., outside the interquartile range). Source data are available online for this figure.

 © The Author(s)

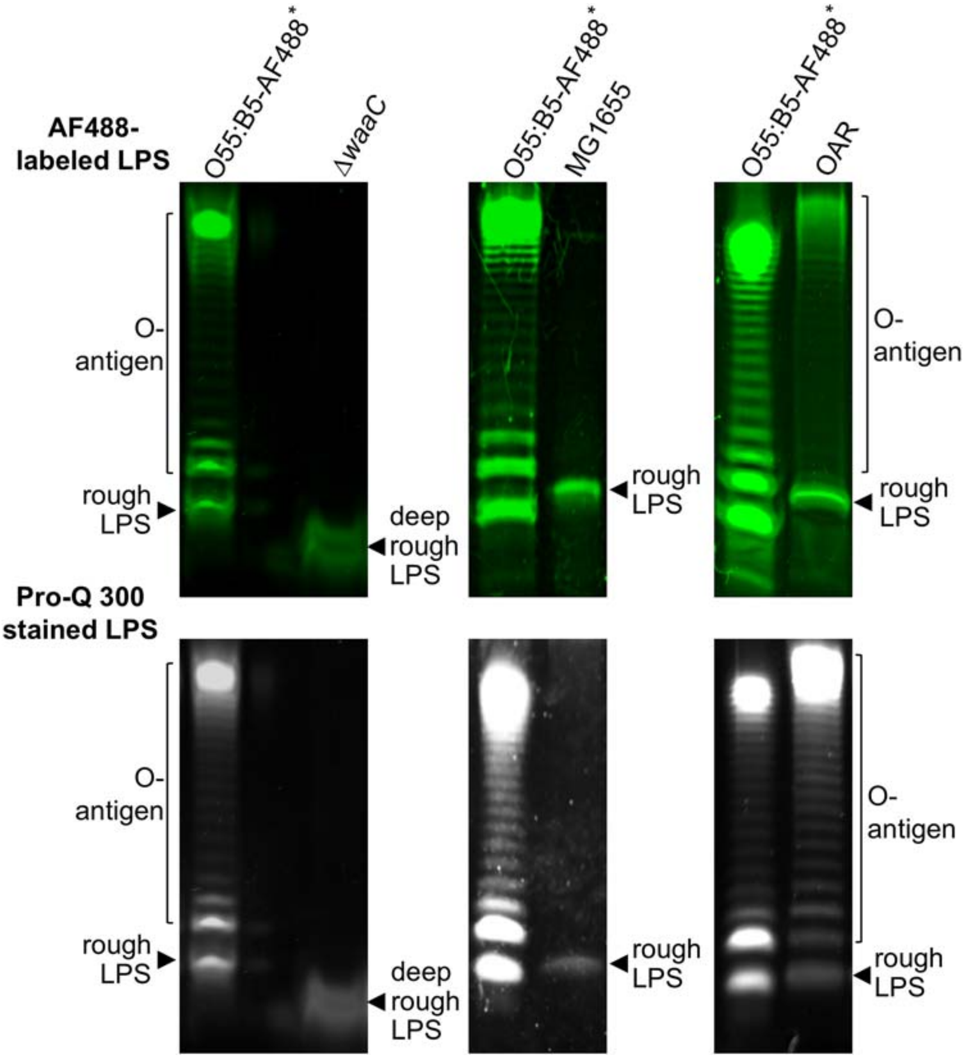

Figure EV3. TSDS-PAGE analysis of LPS extracted from bacterial cells after two-step metabolic / bio-orthogonal labeling with Kdo-N_3 and AF488-alkyne.

LPS was extracted from *E. coli* Δ*waaC* (left-hand gel images), *E. coli* MG1655 (middle gel images) and *E. coli* DFB1655 O-antigen restored (OAR) cells (right-hand gel images). The *E. coli* mutant strains (Δ*lpp*, Δ*ompA*) are in a BW25113 background. LPS extracted from the wild-type BW25113 strain yields the same TSDS-PAGE result as MG1655. AF488-labeled LPS was visualized with 488 nm laser excitation (top row of gel images) and total LPS was visualized using Pro-Q Emerald 300 LPS staining with UV illumination (bottom row of gel images). *Purchased AF488-labeled O55:B5 LPS standard (0.1 µg per lane). Source data are available online for this figure.

© The Author(s)

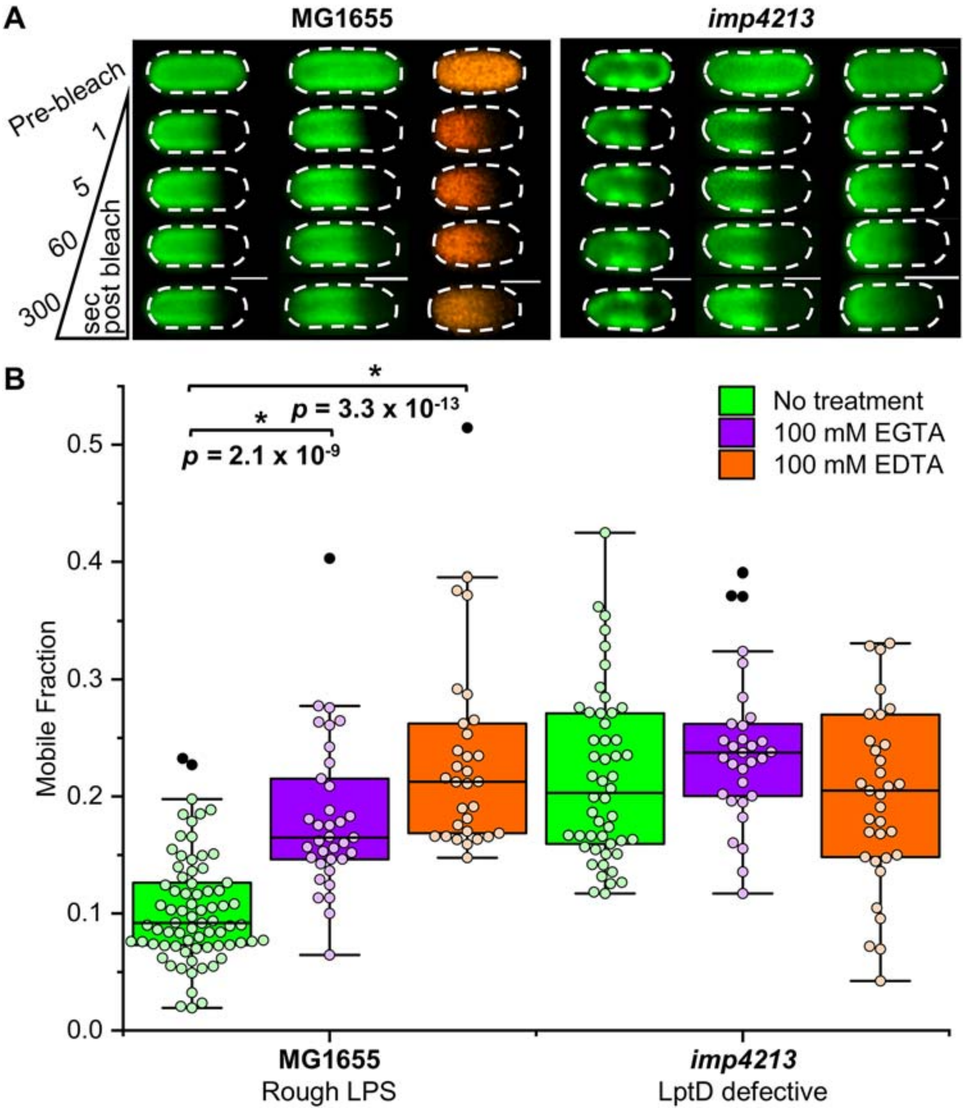

Figure EV4. Observed differences in the sensitivity of LPS mobility to chelator treatments for *E. coli* MG1655 and *imp4213* cells suggests divalent cation-mediated LPS–LPS interactions contribute to LPS restriction in the OM.

(**A**) Representative FRAP sequences for AF488-LPS (green) or AF568-LPS (orange) in the OM of *E. coli* MG1655 and *imp4213* cells without (left-hand vertical image sequence) or with 100 mM EGTA (middle vertical image sequence) and 100 mM EDTA (right-hand vertical image sequence) treatments. Dashed border denotes the outline of each bacterial cell. Scale bars: 1.0 µm. (**B**) Effect of 100 mM EGTA and 100 mM EDTA treatments on LPS mobile fraction distributions in the OM of *E. coli* MG1655 and *imp4213* cells measured via FRAP. The FRAP sequences and mobile fraction data for the *E. coli* MG1655 strain were reused from Fig. 4A,B. All FRAP experiments were done in triplicate using independent biological samples. For each box plot, the center line is the median while the box defines the upper and lower quartiles of the data and the whiskers enclose the interquartile range (i.e., middle 50% of the data). Each symbol represents an individual measurement with black-filled symbols classified as outliers (i.e., outside the interquartile range). MG1655: no treatment, median = 0.092 ($n = 74$); 100 mM EGTA, median = 0.165 ($n = 35$); 100 mM EDTA, median = 0.213 ($n = 31$). *imp4213*: no treatment, median = 0.207 ($n = 49$); 100 mM EGTA, median = 0.237 ($n = 31$); 100 mM EDTA, median = 0.205 ($n = 35$). The disruption of divalent-cation-mediated LPS-LPS interactions by chelator treatments is responsible for the observed significant increase (by Mann–Whitney test) in LPS mobile fractions for the MG1655 cells. In contrast, the disruption of OM asymmetry in the *imp4213* cells caused by phospholipid flipping into the outer leaflet of the OM appears to disrupt these divalent cation-mediated interactions yielding a much lower overall sensitivity to chelator treatment for this strain. Source data are available online for this figure.

 © The Author(s)
